# Analysis of Discrepancies Between Pulse Oximetry and Arterial Oxygen Saturation Measurements by Race and Ethnicity and Association With Organ Dysfunction and Mortality

**DOI:** 10.1001/jamanetworkopen.2021.31674

**Published:** 2021-11-03

**Authors:** An-Kwok Ian Wong, Marie Charpignon, Han Kim, Christopher Josef, Anne A. H. de Hond, Jhalique Jane Fojas, Azade Tabaie, Xiaoli Liu, Eduardo Mireles-Cabodevila, Leandro Carvalho, Rishikesan Kamaleswaran, R. W. M. A. Madushani, Lasith Adhikari, Andre L. Holder, Ewout W. Steyerberg, Timothy G. Buchman, Mary E. Lough, Leo Anthony Celi

**Affiliations:** 1 Division of Pulmonary, Allergy, Critical Care, and Sleep Medicine, Emory University, Atlanta, Georgia; 2 Division of Pulmonary, Allergy, and Critical Care Medicine, Duke University, Durham, North Carolina; 3 MIT Institute for Data, Systems and Society, Cambridge, Massachusetts; 4 Department of Biomedical Engineering, Johns Hopkins University, Baltimore, Maryland; 5 Department of Surgery, Emory University, Atlanta, Georgia; 6 Leiden University Medical Centre, Department of Biomedical Data Sciences, Leiden, the Netherlands; 7 Leiden University Medical Centre, Department of Information Technology and Digital Innovation, Leiden, the Netherlands; 8 Department of Neurology, Beth Israel Deaconess Medical Center, Harvard Medical School, Boston, Massachusetts; 9 Department of Biomedical Informatics, Emory University, Atlanta, Georgia; 10 School of Biological Science and Medical Engineering, Beihang University, Beijing, China; 11 Respiratory Institute, Cleveland Clinic, Cleveland, Ohio; 12 Sociedade Mineira de Terapia Intensiva, Belo Horizonte, Brazil; 13 Department of Infectious Diseases, Boston Medical Center, Boston, Massachusetts; 14 Connected Care and Personal Health, Philips Research North America, Cambridge, Massachusetts; 15 Department of Surgery, Emory University, Atlanta, Georgia; 16 Medicine–Primary Care and Population Health, Stanford University, California; 17 Office of Research, Stanford Health Care, Stanford, California; 18 Massachusetts Institute of Technology, Laboratory for Computational Physiology, Cambridge; 19 Division of Pulmonary, Critical Care, and Sleep Medicine, Beth Israel Deaconess Medical Center, Boston, Massachusetts; 20 Department of Biostatistics, Harvard T.H. Chan School of Public Health, Boston, Massachusetts

## Abstract

**Importance:**

Discrepancies in oxygen saturation measured by pulse oximetry (Spo_2_), when compared with arterial oxygen saturation (Sao_2_) measured by arterial blood gas (ABG), may differentially affect patients according to race and ethnicity. However, the association of these disparities with health outcomes is unknown.

**Objective:**

To examine racial and ethnic discrepancies between Sao_2_ and Spo_2_ measures and their associations with clinical outcomes.

**Design, Setting, and Participants:**

This multicenter, retrospective, cross-sectional study included 3 publicly available electronic health record (EHR) databases (ie, the Electronic Intensive Care Unit–Clinical Research Database and Medical Information Mart for Intensive Care III and IV) as well as Emory Healthcare (2014-2021) and Grady Memorial (2014-2020) databases, spanning 215 hospitals and 382 ICUs. From 141 600 hospital encounters with recorded ABG measurements, 87 971 participants with first ABG measurements and an Spo_2_ of at least 88% within 5 minutes before the ABG test were included.

**Exposures:**

Patients with hidden hypoxemia (ie, Spo_2_≥88% but Sao_2_ <88%).

**Main Outcomes and Measures:**

Outcomes, stratified by race and ethnicity, were Sao_2_ for each Spo_2_, hidden hypoxemia prevalence, initial demographic characteristics (age, sex), clinical outcomes (in-hospital mortality, length of stay), organ dysfunction by scores (Sequential Organ Failure Assessment [SOFA]), and laboratory values (lactate and creatinine levels) before and 24 hours after the ABG measurement.

**Results:**

The first Spo_2_-Sao_2_ pairs from 87 971 patient encounters (27 713 [42.9%] women; mean [SE] age, 62.2 [17.0] years; 1919 [2.3%] Asian patients; 26 032 [29.6%] Black patients; 2397 [2.7%] Hispanic patients, and 57 632 [65.5%] White patients) were analyzed, with 4859 (5.5%) having hidden hypoxemia. Hidden hypoxemia was observed in all subgroups with varying incidence (Black: 1785 [6.8%]; Hispanic: 160 [6.0%]; Asian: 92 [4.8%]; White: 2822 [4.9%]) and was associated with greater organ dysfunction 24 hours after the ABG measurement, as evidenced by higher mean (SE) SOFA scores (7.2 [0.1] vs 6.29 [0.02]) and higher in-hospital mortality (eg, among Black patients: 369 [21.1%] vs 3557 [15.0%]; *P* < .001). Furthermore, patients with hidden hypoxemia had higher mean (SE) lactate levels before (3.15 [0.09] mg/dL vs 2.66 [0.02] mg/dL) and 24 hours after (2.83 [0.14] mg/dL vs 2.27 [0.02] mg/dL) the ABG test, with less lactate clearance (−0.54 [0.12] mg/dL vs −0.79 [0.03] mg/dL).

**Conclusions and Relevance:**

In this study, there was greater variability in oxygen saturation levels for a given Spo_2_ level in patients who self-identified as Black, followed by Hispanic, Asian, and White. Patients with and without hidden hypoxemia were demographically and clinically similar at baseline ABG measurement by SOFA scores, but those with hidden hypoxemia subsequently experienced higher organ dysfunction scores and higher in-hospital mortality.

## Key Points

**Question:**

Do pulse oximetry discrepancies, hidden hypoxemia, and clinical outcomes differ among racial and ethnic subgroups?

**Findings:**

In this cross-sectional study of 5 databases with 87 971 patients, significant disparities in pulse oximetry accuracy across racial and ethnic subgroups (ie, Asian, Black, Hispanic, and White individuals) were found, with higher rates of hidden hypoxemia associated with mortality, future organ dysfunction, and abnormal laboratory test results.

**Meaning:**

In this study, discrepancies in pulse oximetry accuracy among racial and ethnic subgroups were associated with higher rates of hidden hypoxemia, mortality, and organ dysfunction.

## Teaser

This cross-sectional study examines racial and ethnic discrepancies between oxygen saturation measured by pulse oximetry and arterial blood gas and their associations with clinical outcomes.

## Introduction

Recently, reports of systemic racial bias in which oxygen saturation measured by pulse oximeter (Spo_2_) overestimates the true arterial oxygen saturation (Sao_2_) in patients with darkly pigmented skin has raised concerns about the clinical accuracy of pulse oximetry.^1^ Sjoding et al^1^ used race listed in the EHR as a proxy for skin color to retrospectively analyze the accuracy of pulse oximetry during hypoxemia in 2 high-acuity adult cohorts. They described occult hypoxemia as an Sao_2_ of less than 88% when the Spo_2_ was between 92% and 96%. In both cohorts, the incidence of hidden hypoxemia was almost 3 times higher among patients self-reported as Black vs White.^1^

Pulse oximetry is a useful tool to monitor blood oxygen saturation without obtaining an invasive arterial blood gas (ABG) measurement. The US Food and Drug Administration (FDA) requires root mean square accuracy within 2% for values between 70% and 100%, implying that an adequate pulse oximeter returns an Spo_2_ value within 2% to 3% of the Sao_2_ value (ie, a range of 4%-6%) only two-thirds of the time.^2^

Studies have highlighted the inaccuracy of pulse oximetry in critically ill patients; however, smaller sample sizes hindered in-depth analysis of race, ethnicity, and outcomes (eTable 1 in the Supplement).^3,4,5,6,7^ This study used 5 large EHR data sets of critically ill patients to further evaluate the incidence and clinical outcomes of hidden hypoxemia across racial and ethnic groups.

## Methods

This study followed the Strengthening the Reporting of Observational Studies in Epidemiology (STROBE) reporting guideline.^8^ Data in Medical Information Mart for Intensive Care III (MIMIC-III), MIMIC-IV, and Electronic Intensive Care Unit–Clinical Research Database (eICU-CRD) had been previously deidentified and did not require a waiver for informed consent. The Medical Information Mart for Intensive Care (MIMIC) database is a collaboration between the Beth Israel Deaconess Medical Center and the Laboratory for Computational Physiology at the Massachusetts Institute of Technology. The database contains granular, deidentified ICU data from the Beth Israel Deaconess Medical Center. The data have been generated from more than 70 intensive care unit beds with medical, surgical, cardiac, and neurological patients. We used the latest data version, MIMIC-III (version 1.4), which contains deidentified data associated with 53 423 ICU admissions.^9^ Physionet approved the use of MIMIC for this study. Emory University approved the use of the Emory and Grady databases for research, with a complete HIPAA and informed consent waiver.

### Data Sources

#### Open Source PhysioNet Databases

The eICU-CRD (comprising 335 ICUs across 208 hospitals) and MIMIC databases (comprising 6 ICUs in 1 hospital) were used. The Sequential Organ Failure Assessment (SOFA) scores (eTable 2 in the Supplement), including the cardiovascular SOFA (CVSOFA) and respiratory SOFA (RSOFA) scores, were provided by MIT Laboratory of Computational Physiology.^9,10,11,12,13^

#### Emory Healthcare and Grady Memorial

Data were collected from all units in Emory Healthcare (277 units, including 26 ICUs, across 4 hospitals) and Grady Memorial Hospital (73 units, including 9 ICUs, in 1 hospital). Emory Healthcare and Grady patient data spanned 2014 to 2021 and 2014 to 2020, respectively. SOFA scores and its components were not available for the Emory and Grady databases.

### Data Extraction

Data analysis was conducted with R version 3.6.3 (R Project for Statistical Computing) and Python version 3.6 (Python Software Foundation). All ABG and Spo_2_ values were extracted from the EHR.

#### Inclusion and Exclusion Criteria

An Spo_2_ range of 88% to 100% was selected as an interval in which patients may have hypoxemia but falsely be considered as having arterial blood oxygenation in the reference range according to Sao_2_. Each ABG-measured Sao_2_ was matched with the closest Spo_2_ value recorded within the previous 5 minutes. To eliminate repeated measurements and limit confounding, only the first ABG measurement from each hospital encounter was used. Spo_2_ measurements of less than 88% were not examined because of low prevalence in the EHR.

#### Race and Ethnicity

In each EHR data set, race and ethnicity were defined using self-reported demographic data, including administrative entries with additional identifiers. All patients with race and ethnicity information who could not be classified as Asian, Black, Hispanic, or White were excluded. Patients were stratified by age, sex, race and ethnicity, and CVSOFA score. If any of these characteristics were missing, a patient was excluded from the corresponding subgroup analysis but included in the overall analysis.

### Statistical Analysis

For each racial and ethnic group, the frequency of ABG measurement was characterized using 2 complementary analyses. First, encounters with ABG measurements were compared with encounters without ABGs measurements to determine the likelihood of receiving an ABG measurement during an encounter by race and ethnicity. Second, to characterize the rate of ABG collection across encounters with at least 1 ABG, the total number of ABGs normalized by the length of stay (in days) was calculated. Given possible confounding by illness severity, the second estimates were stratified by CVSOFA score at the time of ABG.

Differences in Spo_2_-Sao_2_ pairs were characterized by modified Bland-Altman plots. We used χ^2^ tests to compare the distribution of categorical variables (eg, sex) between any 2 groups, while Mann-Whitney nonparametric tests were used for continuous and ordinal variables (eg, age, SOFA score). Notably, differences in the distribution of numeric clinical end points (eg, SOFA score) were evaluated via bootstrapping (100 iterations), followed by a Mann-Whitney nonparametric test. Differences between stratified odds differences (eg, risk of hidden hypoxemia by race and ethnicity) were tested with the Breslow-Day test.

Hidden hypoxemia was defined as an Spo_2_of88% or greater despite an Sao_2_ of less than 88%. Patients with and without hidden hypoxemia were compared at the time of ABG measurement by baseline demographic characteristics (age, sex) and by organ failure scores (SOFA, RSOFA, CVSOFA). The long-term association of hidden hypoxemia with clinical outcomes was analyzed by estimating differences in length of stay and in-hospital mortality. The short-term association of hidden hypoxemia with organ dysfunction was examined using SOFA and CVSOFA scores measured 24 hours after the baseline ABG measurement. Associations between hidden hypoxemia, RSOFA, and in-hospital mortality were also examined. The consequences of hidden hypoxemia were also evaluated by comparing the last serum lactate and serum creatinine levels in a 7-day window before the ABG measurement with the first value in a 7-day window starting 24 hours after the ABG measurement. Values were compared at each time in addition to the difference between the before and after values.

Multivariate logistic regression was used for assessing binary end points (eg, in-hospital mortality), multivariate ordinal regression for numerical end points (eg, CVSOFA and RSOFA scores), and multivariate linear models for continuous end points (eg, creatinine lactate levels), using analysis of variance to test for the impact of hidden hypoxemia while adjusting for other covariates (eg, age, sex, SOFA score at time of ABG measurement) (eAppendix 1 in the Supplement). Missing data for any covariates were flagged with a separate binary variable.

Statistical analysis was conducted with R version 3.6.3 (R Project for Statistical Computing) and Python version 3.6 (Python Software Foundation). Statistical significance was set at *P* < .05, and all tests were 2-tailed.

## Results

**Figure 1.**
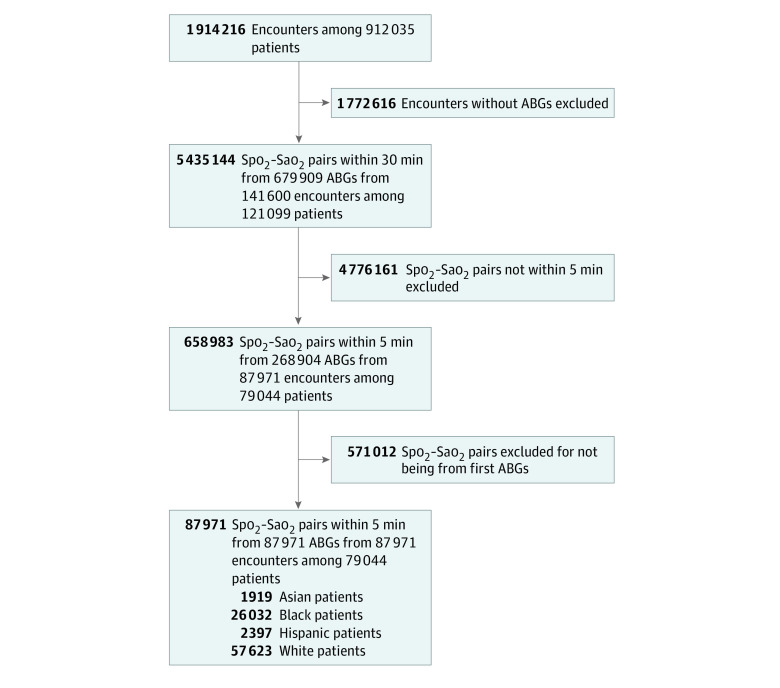
Flow Diagram Aggregate flow diagram for all data sets. ABG indicates arterial blood gas; Sao_2_, arterial oxygen saturation; Spo_2_, oxygen saturation by pulse oximetry.

The first Spo_2_-Sao_2_ pairs from 87 971 patient encounters (27 713 [42.9%] women; mean [SE] age, 62.2 [17.0] years) were analyzed among 4 race/ethnicity subgroups (Asian, 1919 patients [2.3%]; Black, 26 032 [29.6%]; Hispanic, 2397 patients [2.7%]; White, 57 623 patients [65.5%]), with 4859 (5.5%) having hidden hypoxemia. In total, 141 600 patients, with 679 909 ABGs and 5 435 144 Spo_2_-Sao_2_ pairs within 30 minutes of each other were identified. Patient characteristics are presented in Table 1 and Figure 1. Restricting to Spo_2_ measurements to those within the 5 minutes preceding the ABG measurement resulted in 268 904 Spo_2_-Sao_2_ pairs; further selecting the first ABG in an encounter led to 87 971 Spo_2_-Sao_2_ pairs (eAppendix 2 in the Supplement). Sample sizes were considerably smaller for Asian and Hispanic subgroups.

**Table 1.** Characteristics of Patients with Spo_2_ of at Least 88% 5 Minutes Before First ABG Measurement^a^

Characteristic	Patients, No. (%)
Total, No.	
Patients	79 044
Hospital encounters	87 971
ABG	87 971
Spo_2_-Sao_2_ pairs	87 971
Sex	
Female	37 713 (42.9)
Male	50 258 (57.1)
Age, mean (SE), y	62.18 (16.97)
Sao_2_, mean (SE), %	95.33 (0.03)
Spo_2_, mean (SE), %	97.12 (0.01)
Location	
ICU	75 397 (85.7)
ED	6071 (6.9)
Stepdown	570 (0.6)
Floor	3193 (3.6)
PACU	542 (0.6)
OR	1112 (1.3)
Other or unknown	1086 (1.2)
Race and ethnicity	
Asian	1919 (2.2)
Black	26 032 (29.6)
Hispanic	2397 (2.7)
White	57 623 (65.5)
Database	
eICU-CRD	38 693 (44.0)
MIMIC-III	2017 (2.3)
MIMIC-IV	4353 (5.0)
Emory	33 157 (37.7)
Grady	9751 (11.1)
Cardiovascular SOFA score, mean (SE)^b^	0.67 (0.006)

Abbreviations: ABG, arterial blood gas; ED, emergency department; eICU-CRD, Electronic Intensive Care Unit–Clinical Research Database; ICU, intensive care unit; MIMIC, Medical Information Mart for Intensive Care; OR, operating room; PACU, postanesthesia care unit; Sao_2_, arterial oxygen saturation; SOFA, Sequential Organ Failure Assessment; Spo_2_, oxygen saturation by pulse oximeter.

^a^ Table presents patient characteristics for all ABGs examined, which represents characteristics of patients with an Spo_2_ within the 5 minutes preceding the ABG, based on the first ABG measurement of their hospital encounter. When applicable, SEs are provided. They were obtained using simple bootstrap with 100 iterations.

^b^ Cardiovascular SOFA scores available only for patients in eICU-CRD, MIMIC-III, and MIMIC-IV databases.

### Missingness

Missing data on length of stay and in-hospital mortality was 0.1% (96 encounters) and 0.4% (369 encounters), respectively. Given that there were no SOFA score data from Emory and Grady, RSOFA, CVSOFA, and SOFA scores had 52.7% (46 381 encounters), 52.9% (46 538 encounters), and 52.7% (46 381 encounters) missingness, respectively. Missingness for Spo_2_ variability and other variables are described in eTable 4 and eTable 5 in the Supplement.

### ABG Measurement Frequency by Race and Ethnicity

There were differences in the likelihood of receiving an ABG measurement during a hospital encounter that varied by race and ethnicity, with the White subgroup most likely to receive an initial ABG measurement despite similar RSOFA and CVSOFA scores: White patients, 85 872 of 1 532 492 encounters (5.6%); Asian patients, 3249 of 95 813 (3.4%); Black patients, 49 053 of 1 781 868 (2.8%); and Hispanic patients, 3426 of 179 617 (1.9%) (eTable 6 in the Supplement). Although following the first ABG, subsequent ABG frequencies were similar across racial and ethnic subgroups (eTable 7 in the Supplement). A table comparing patients with and without ABG measurements across races and ethnicities appears in eTable 3 in the Supplement.

### Comparison of Spo_2_ With Sao_2_ by Race and Ethnicity

The White subgroup was used as a reference because it had the highest prevalence in the data set. At most Spo_2_ values, there were statistically significant differences in true oxygen saturation levels between White patients compared with those in other racial and ethnic subgroups, although these differences were small in magnitude (evaluated using means and medians) (eTable 5 in the Supplement). Similar results were obtained when using all ABGs measured during a patient’s hospitalization (268 904) instead of the first ABG measurement only (87 971) (eFigure 1 in the Supplement). This finding was robust to adjustment for sex, age, and RSOFA and CVSOFA scores (eFigures 2-5 in the Supplement).

### Hidden Hypoxemia by Race and Ethnicity

**Figure 2.**
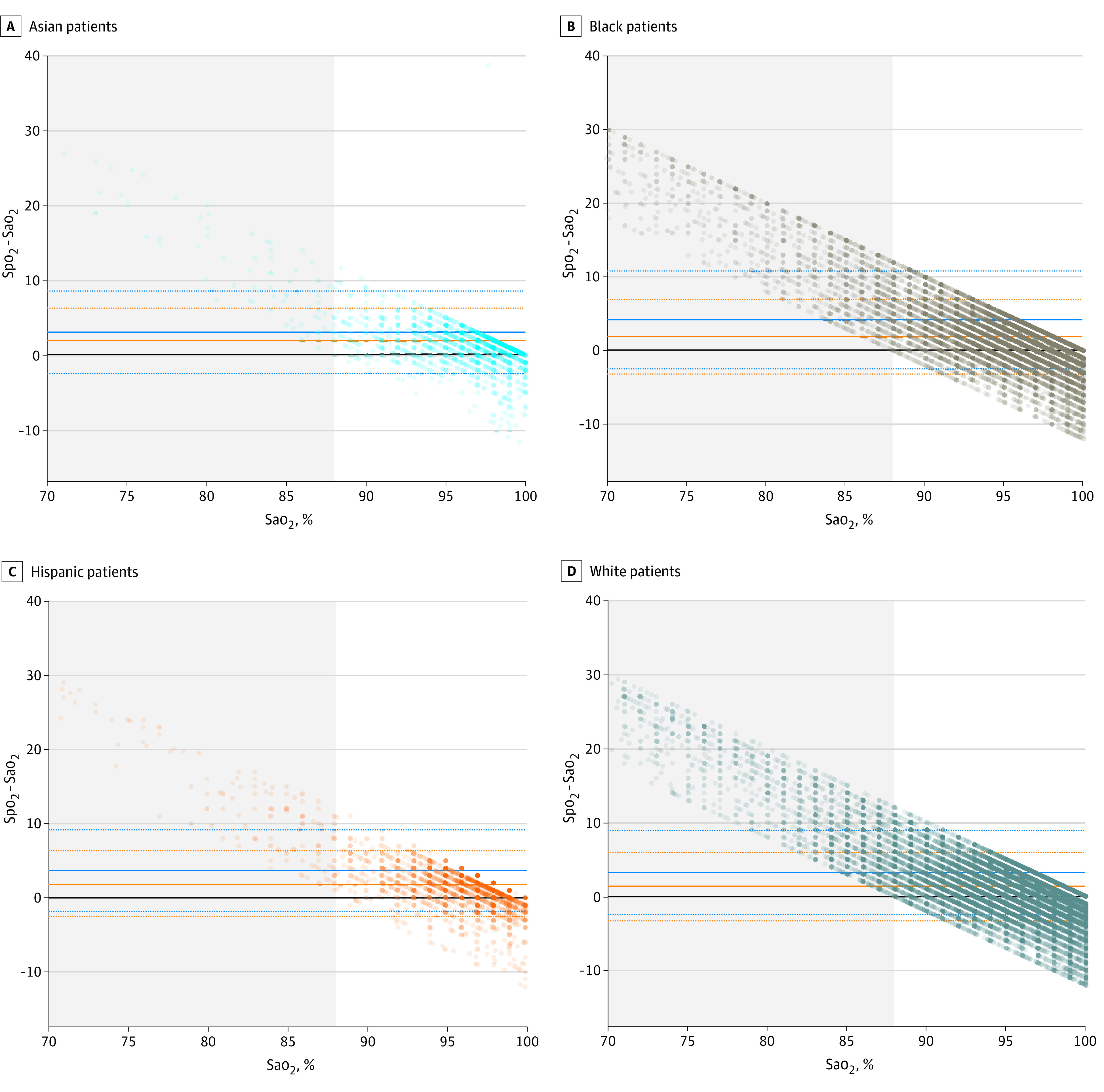
Modified Bland-Altman Plots by Race and Ethnicity On each plot, the bold horizontal lines represent the mean bias (defined as the difference between the oxygen saturation measured by pulse oximetry [Spo_2_] and arterial blood gas value [Sao_2_]) for each of the 2 Spo_2_ groups. The blue lines, with dashed blue lines indicating 95% CIs, are for the group with Spo_2_ of 88% to 92%, and the orange lines, with dashed orange lines indicating 95% CIs, for the group with Spo_2_ of 93% to 96%. The solid black line indicates the absence of bias (ie, Spo_2_ − Sao_2_ = 0). When the mean bias is above the black line, there is positive bias in the pulse oximetry measurement (ie, the observed Spo_2_ is greater than the true Sao_2_). In contrast, when the mean bias is below the black line, there is negative bias in the pulse oximetry measurement (ie, the observed Spo_2_ is below the true Sao_2_).

Hidden hypoxemia occurred across all racial and ethnic subgroups, assessed using the first ABG measurement with an Spo_2_ level greater than 88%. Patients self-identified as Black had higher Sao_2_ variability for any given Spo_2_ value, as evidenced by a larger IQR (eg, median [IQR] Sao_2_ at Spo_2_ of 88%, Black patients: 90.10% [10.13]; White patients, 90.00% [9.10]). There was a varying incidence of hidden hypoxemia among racial and ethnic group in descending order: Black, 1785 [6.8%]; Hispanic, 160 [6.0%]; Asian, 92 [4.8%]; White, 2822 [4.9%] (*P* < .001) (Figure 2; eTable 8 and eTable 9 in the Supplement).

#### Organ Dysfunction at Time of First ABG Measurement and 24 Hours Later

Across all racial and ethnic groups statistically significant, although clinically small, differences in baseline organ dysfunction (SOFA, CVSOFA, RSOFA) were present at the time of the first ABG measurements between patients with and without hidden hypoxemia (Table 2). Furthermore, across all racial and ethnic groups, patients with hidden hypoxemia subsequently experienced greater organ dysfunction than patients without hidden hypoxemia, as evidenced by higher CVSOFA and SOFA scores measured 24 hours after the ABG measurement was drawn (mean [SE] CVSOFA: 1.48 [0.03] vs 1.25 [0.01]; mean [SE] SOFA: 7.2 [0.1] vs 6.29 [0.02]) (Table 2). This difference persisted even when adjusted for age, sex, and SOFA score (data not shown).

**Table 2.** Descriptive Statistics for Patients With Hidden Hypoxemia vs Patients Without Hypoxemia^a^

Characteristic	Asian patients, mean (SE)		*P* value	Black patients, mean (SE)		*P* value	Hispanic patients, mean (SE)		*P* value	White patients, mean (SE)		*P* value
	Hidden hypoxemia	No hypoxemia		Hidden hypoxemia	No hypoxemia		Hidden hypoxemia	No hypoxemia		Hidden hypoxemia	No hypoxemia	
No. (%)	94 (4.9)	1825 (95.1)	NA	1789 (6.9)	24 243 (93.1)	NA	145 (6.0)	2252 (94.0)	NA	2831 (4.9)	54 792 (95.1)	NA
Age, y	63.93 (2.46)	62.34 (0.51)	<.001	58.26 (0.51)	57.76 (0.15)	<.001	57.48 (2.05)	59.53 (0.52)	<.001	64.03 (0.41)	64.29 (0.1)	<.001
Sex, No. (%)												
Female	36 (38.3)	724 (39.7)	.89	862 (48.2)	11 243 (46.4)	.11	75 (51.7)	1028 (45.6)	.15	1241 (43.8)	22 504 (41.1)	.01
Male	58 (61.7)	1101 (60.3)		927 (51.8)	13 000 (53.6)		70 (48.3)	1224 (54.4)		1590 (56.2)	32 288 (58.9)	
Serum creatinine levels, mg/dL												
Before ABG	1.73 (0.24)	1.59 (0.07)	<.001	2.37 (0.09)	2.27 (0.03)	<.001	1.79 (0.19)	1.66 (0.04)	<.001	1.73 (0.04)	1.46 (0.01)	<.001
After ABG	1.79 (0.31)	1.44 (0.06)	<.001	2.2 (0.08)	2.07 (0.02)	<.001	2.01 (0.33)	1.6 (0.07)	<.001	1.53 (0.06)	1.38 (0.01)	<.001
Difference	0.05 (0.19)	−0.1 (0.04)	<.001	−0.21 (0.06)	−0.2 (0.02)	.08	−0.15 (0.19)	−0.17 (0.04)	.33	−0.17 (0.05)	−0.07 (0.01)	<.001
Serum lactate levels, mg/dL												
Before ABG	4.37 (0.82)	2.87 (0.11)	<.001	3.4 (0.13)	2.85 (0.03)	<.001	2.96 (0.45)	2.87 (0.09)	.26	2.99 (0.1)	2.56 (0.02)	<.001
After ABG	2.97 (0.65)	2.32 (0.15)	<.001	3.27 (0.22)	2.5 (0.04)	<.001	3.4 (0.8)	2.24 (0.13)	<.001	2.51 (0.14)	2.15 (0.03)	<.001
Difference,	−1.61 (0.86)	−1.14 (0.2)	<.001	−0.34 (0.25)	−0.82 (0.05)	<.001	−0.41 (0.79)	−1.14 (0.18)	<.001	−0.68 (0.2)	−0.74 (0.04)	.00
Long-term clinical outcomes												
Hospital LOS, d^b^	13.63 (1.81)	13.18 (0.5)	.02	13.51 (0.58)	16.48 (0.18)	.00	14.01 (2.11)	13.18 (0.56)	.00	10.67 (0.35)	11.19 (0.07)	.00
In-hospital death, No. (%)	20 (21.3)	286 (15.7)	.13	369 (20.6)	3557 (14.7)	<.001	35 (24.1)	439 (19.5)	.06	738 (26.1)	8238 (15.0)	<.001
SOFA score^c^												
At the time of ABG												
CVSOFA	0.61 (0.2)	0.73 (0.06)	<.001	0.98 (0.07)	0.85 (0.02)	<.001	1.05 (0.15)	1.03 (0.03)	.14	1.13 (0.03)	0.99 (0.01)	<.001
RSOFA	1.56 (0.32)	1.26 (0.06)	<.001	1.87 (0.12)	1.4 (0.03)	<.001	2.02 (0.17)	1.44 (0.04)	<.001	1.81 (0.04)	1.38 (0.01)	<.001
SOFA	6.03 (0.75)	5.82 (0.15)	<.001	4.98 (0.25)	5.26 (0.06)	<.001	5.09 (0.54)	5.37 (0.09)	<.001	5.27 (0.1)	5.22 (0.03)	<.001
24 h After ABG												
CVSOFA	1.52 (0.2)	1.36 (0.05)	<.001	7.02 (0.29)	6.33 (0.07)	<.001	1.56 (0.15)	1.32 (0.03)	<.001	1.48 (0.03)	1.26 (0.01)	<.001
RSOFA	1.58 (0.28)	1.27 (0.07)	<.001	1.87 (0.1)	1.4 (0.03)	<.001	2.01 (0.18)	1.45 (0.04)	<.001	1.79 (0.04)	1.37 (0.01)	<.001
SOFA	7.17 (0.77)	5.62 (0.17)	<.001	8.17 (0.27)	7.26 (0.08)	<.001	7.49 (0.54)	6.3 (0.1)	<.001	7.2 (0.1)	6.3 (0.02)	<.001

Abbreviations: ABG, arterial blood gas; CVSOFA, cardiovascular Sequential Organ Failure Assessment; LOS, length of stay; NA, not applicable; RSOFA, respiratory Sequential Organ Failure Assessment; SOFA, Sequential Organ Failure Assessment.

SI conversion factors: To convert creatinine to micromoles per liter, multiply by 88.4; lactate to millimoles per liter, multiply by 0.111.

^a^ The table presents baseline patient demographic (age, sex) and clinical characteristics (SOFA, CVSOFA) at the time of ABG, stratified by race and ethnicity. When applicable, SEs are provided. These were obtained using simple bootstrap with 100 iterations.

^b^ Length of stay computed for survivors only.

^c^ SOFA scores available only for patients in the Electronic Intensive Care Unit–Clinical Research Database and Medical Information Mart for Intensive Care databases.

#### Clinical Outcomes

Across all racial and ethnic groups, patients with hidden hypoxemia had higher in-hospital mortality than patients without hidden hypoxemia (Table 2). The difference was significant for Black and White patients (eg, Black patients: 369 [21.1%] vs 3557 [15.0%]; *P* < .001). This association persisted even when adjusted for age, sex, SOFA score. However, there was no differences in length of stay for patients with and without hidden hypoxemia when considering survivors only (Table 2).

Across all racial and ethnic subgroups, patients with hidden hypoxemia had a significantly higher mean (SE) serum creatinine level (1.96 [0.04] mg/dL vs 1.69 [0.01] mg/dL [to convert to micromoles per liter, multiply by 88.4]; *P* < .001) and serum lactate level (3.15 [0.09] mg/dL vs 2.66 [0.02] mg/dL [to convert to millimoles per liter, multiply by 0.111]; *P* < .001) before the ABG measurement than patients without hypoxemia. Patients with hidden hypoxemia continued to maintain significantly higher mean (SE) serum creatinine (1.86 [0.05] mg/dL vs 1.63 [0.01] mg/dL; *P* < .001) and lactate (2.83 [0.14] mg/dL vs 2.83 [0.14] mg/dL; *P* < .001) values after the ABG measurements. When comparing values before and after the ABG, serum lactate demonstrated a smaller mean (SE) decrease among patients with hidden hypoxemia overall (−0.54 [0.12] vs −0.79 [0.03]; *P* < .001) and in all racial and ethnic groups except Asian patients. However, the difference in serum creatinine levels before and after the ABG measurement was not consistent across race and ethnicity and hidden hypoxemia status.

#### Hidden Hypoxemia and RSOFA Score

The likelihood of hidden hypoxemia increased with higher RSOFA values and was highest among patients with the highest RSOFA scores at baseline (ie, at the time of ABG) (eTable 10 in the Supplement). The presence of hidden hypoxemia, irrespective of RSOFA score, was associated with higher risk of mortality (eTable 11 in the Supplement).

### Risk of Hidden Hypoxemia by Race and Ethnicity

Hidden hypoxemia occurred across racial and ethnic groups, but the risk differed among subgroups when calculated with a risk-threshold of 5%. White patients had a 5% risk of hidden hypoxemia at an Spo_2_ of 94%, whereas this 5% risk occurred at a higher Spo_2_ for Black (97%), Hispanic (97%), and Asian (95%) patients. The risk of hidden hypoxemia at an SpO_2_ of 93% to 96% is 6.5% in White patients, increasing to 6.6% for Hispanic patients and up to 10.9% for Black and Asian patients (a 68% higher relative risk). In conjunction with eTable 8 in the Supplement, which shows more granular data, a clinician could select their threshold for an acceptable risk of hidden hypoxemia by Spo_2_ target by selecting the highest Spo_2_ with a fixed risk of hidden hypoxemia. To ensure a risk of hidden hypoxemia of less than 10%, Spo_2_ should be greater than 93% among Asian patients (mean [SE] risk, 11.0% [5.0]), 96% among Black patients (mean [SE] risk, 8.2% [1.0]); 92% among Hispanic patients (mean [SE] risk, 18.4% [6.0]), and 93% among White patients (mean [SE] risk, 10.6% [1.0]) (Table 3).

**Table 3.** Risk of Hidden Hypoxemia by Race and Ethnicity^a^

Spo_2_ group, %	Asian patients		Black patients		Hispanic patients		White patients	
	Mean (SE) %	No.	Mean (SE) %	No.	Mean (SE) %	No.	Mean (SE) %	No.
88-92	24.2 (5.0)	150	26.1 (1.0)	1993	28.3 (4.0)	182	22.8 (1.0)	5304
93-96	6.8 (2.0)	412	10.9 (1.0)	5454	6.6 (1.0)	594	6.5 (0.0)	15 893
97-100	2.6 (1.0)	1357	4.3 (0.0)	18 585	4.5 (1.0)	1621	2.7 (0.0)	36 426

Abbreviations: Sao_2_, oxygen saturation; Spo_2_, pulse oximeter.

^a^ The risk of hidden hypoxemia (ie, Spo_2 _≥88%; Sao_2 _<88%) characterized by race and ethnicity. Each cell in the table represents the percentage of patients with hidden hypoxemia in the considered racial and ethnic subgroup for a given pulse oximetry grouping. The corresponding bootstrapped SE of the mean for 100 iterations is also provided. The number indicates the total number of patients in each subgroup at that given pulse oximetry grouping (with and without hidden hypoxemia).

## Discussion

To our knowledge, this study is the first to characterize the prevalence of hidden hypoxemia (Sao_2_ <88%, but Spo_2_ ≥88%) by race and ethnicity in hospitalized patients’ first ABG measurements in their hospitalization across 5 large US databases. Racial and ethnic disparities in the incidence of hidden hypoxemia in the hospital are worrying because low oxygen saturation levels, when undetected, can lead to complications in the short and long term.

This analysis demonstrates that all racial and ethnic groups experienced discrepancies in pulse oximetry resulting in hidden hypoxemia; however, it was more prevalent in Asian, Black, and Hispanic patients than White patients. There is a risk of hidden hypoxemia at all Spo_2_ values, and this risk increases as Spo_2_ approaches 88%. When hidden hypoxemia occurs, despite similar organ dysfunction scores at the time of the ABG measurement, it was associated with increased in-hospital mortality, increased short-term future organ dysfunction (ie, SOFA, RSOFA, and CVSOFA scores), and increased laboratory findings (ie, lactate and creatinine levels), even when adjusting for covariates, such as age, sex, and SOFA score. These continue to hold true when restricted to ABGs with carboxyhemoglobin and methemoglobin levels less than 2% (eTable 12 in the Supplement). Furthermore, the presence of hidden hypoxemia was independently associated with increased mortality for all RSOFA values, suggesting that hidden hypoxemia and RSOFA are complementary. Finally, to maintain a risk of hidden hypoxemia less than 10%, each race and ethnicity would have a different Spo_2_ threshold: Asian, 93%; Black, 95%; Hispanic, 92%, White, 93%) (Table 3; eTable 9 in the Supplement).

As this analysis was restricted to the first ABG measurement in a hospitalization, the clinician could not have been aware of hidden hypoxemia prior to the ABG test. It is therefore unknowable how long a patient was truly hypoxemic before their ABG measurement, although the clinician would be aware of hypoxemia once they had the ABG results. Despite similar organ dysfunction scores at the time of the ABG measurement, patients with hidden hypoxemia had greater laboratory abnormalities (ie, for lactate and creatinine levels) before the ABG measurement that persisted for at least 24 hours, suggesting that these patients may have more severe illness. This study was not designed to assess causality; it is both plausible that the patient’s illness could be causative of hidden hypoxemia (and thus be a marker of dysfunction) and that hidden hypoxemia for an unknown (perhaps prolonged) duration resulted in worse organ dysfunction.

The effects of hypoxia can be organized by the duration a patient experiences hypoxia. Brief, acute episodes of hypoxia have been associated with electrocardiogram changes^14^ and, in healthy patients, brief cognitive impairment without sustained long-term cognitive changes.^15^ However, hypoxia has been associated with increased oxidative stress, reactive-oxygen species, angiogenesis, hypoxia-inducible factors, and systemic and vascular inflammation with endothelial dysfunction.^16,17,18^ Critically ill patients may have impaired tolerance of these changes,^17^ and hypoxia can result in kidney injury and lactic acidosis.^19,20,21^

Hidden hypoxemia was prevalent across all racial and ethnic subgroups, but it disproportionately affected certain groups because pulse oximeters are not tested or calibrated on an adequate number of individuals with varying skin pigmentation. Since 2013, the FDA has required that the test sample for pulse oximeters include at least 15% people with diverse skin pigmentation, including 2 individuals with darkly pigmented skin.^22^ However, this sampling does not reflect the United States 2010 census of Asian (5.9% of population), Black (13.4%), Hispanic (18.5%), and White (60.1%) individuals.^23,24^ Going forward, population differences will only increase in relevance as the United States becomes more racially and ethnically diverse.^25^ Furthermore, although older studies questioned the accuracy of pulse oximetry in critical illness, sample sizes were too small to examine the issue of skin color and race and ethnicity in meaningful detail (eFigure 6 in the Supplement).^3,4,5,6,7^

The results of this study highlight several societal and medical issues. First, pulse oximeters are inadequately tested or calibrated in hospitalized patients, despite often being the intended population. Second, a 2% accuracy range (4% total) is too wide at low blood oxygenation levels, and third, pulse oximeters are insufficiently tested across different racial and ethnic groups prior to approval by the FDA. As the results of this study demonstrate, this combination has unintended negative health outcomes. By providing a data-driven approach to identify hidden hypoxemia using pulse oximetry, this study is a step toward greater health care equity.

While a short-term solution to hidden hypoxemia may be to more frequently sample ABG values, such a strategy is invasive and inefficient.^26^ If anything, greater ABG sampling is merely a stopgap to cover the use of imperfect medical technology. The important message is that health care devices, like predictive algorithms and medications, must be designed more inclusively to achieve comparable measurement accuracy irrespective of race and ethnicity. As noted in previous studies,^1,27,28^ pulse oximetry devices are not reliably accurate and do not capture blood oxygenation readings equally across different skin colors. In the meantime, prudent clinicians should note the Spo_2_ reading at the time the ABG is drawn to accurately identify any Spo_2_-to-Sao_2_ discrepancy once the ABG result is reported.

It is important to be cognizant of the patient population in which pulse oximeters used in critical care are validated. There is a need for more transparency in the labeling of all patient care devices, including the detailed characteristics of groups on which they were evaluated. To further achieve more equitable health outcomes, we call for reinforced testing and recalibration of health care devices—across all target patient populations.

### Limitations

This retrospective EHR analysis has inherent limitations that were systematically addressed. First, Sao_2_-Spo_2_ pairs combine measurements that are not always collected simultaneously. Analysis was thus restricted to Spo_2_ values recorded in the 5 minutes preceding the ABG test. Shock and critical illness, in conjunction with other comorbid conditions (eg, peripheral arterial disease, diabetes), may further affect pulse oximetry accuracy and need further characterization for proper adjustment for confounding beyond CVSOFA score.

There was high missingness, especially in SOFA scores and laboratory values, that was accounted for with missing flags during regression analysis; SOFA scores were only calculated for eICU-CRD, MIMIC-III, and MIMIC-IV data and were not calculated for Emory or Grady data. Multiple imputation methods could improve robustness. Additionally, the EHR data did not record Spo_2_ signal quality or pulse oximeter brand, leading to unclear knowledge of Spo_2_ accuracy and homogeneity.

There may be a selection bias in acquiring ABG measurements. Given similar SOFA scores at the time of testing, White patients were significantly more likely to receive the criterion-standard test. It is plausible that there was selection bias with underdetection of hidden hypoxemia among Asian, Black, and Hispanic patients. The disparities in clinical outcomes may be underestimated if more ABG tests were performed in these racial and ethnic subgroups. Additionally, these retrospective analyses reflect associations; future studies should be designed to assess causality.

## Conclusions

In this study, all racial and ethnic subgroups experienced high variability in arterial oxygen saturation for fixed pulse oximetry levels, with a greater discrepancy in patients self-reporting as Asian, Black, and Hispanic than White. Small but statistically significant differences in the bias of Spo_2_ measurements (vs true Sao_2_ measurements) were associated with increased incidence of hidden hypoxemia (Sao_2_<88% despite Spo_2_ ≥88%). Although demographically and clinically similar to patients without hypoxemia at baseline ABG measurement, those with hidden hypoxemia had higher rates of organ dysfunction 24 hours later and higher in-hospital mortality. Validation of all health technologies, including pulse oximetry, must be performed across a wider range of patient populations to avoid perpetuating harm from miscalibration.^22^

## Notes

Supplement. **eAppendix 1.** Supplementary Methods **eAppendix 2.** Supplementary Results **eFigure 1.** Spo_2_-Sao_2_ by Race and Ethnicity, for All ABG Measurements Taken Throughout 268 904 Hospital Encounters **eFigure 2.** Spo_2_-Sao_2_ by Race and Ethnicity, for the First ABG Measurement per 87 971 Hospital Encounters **eFigure 3.** Spo_2_-Sao_2_ by Race and Ethnicity, for the First ABG Measurement per 87 971 Hospital Encounters, Stratified by Age Group **eFigure 4.** Spo_2_-Sao_2_ by Race and Ethnicity, for the First ABG Measurement per 87 971 Hospital Encounters, Stratified by Sex **eFigure 5.** Spo_2_-Sao_2_ by Race and Ethnicity, for the First ABG Measurement per 87 971 Hospital Encounter, Stratified by Cardiovascular SOFA Score **eFigure 6.** Directed Acyclic Graph for Race and Ethnicity, Hidden Hypoxemia, Organ Dysfunction, and Mortality **eTable 1. **Literature Review **eTable 2.** SOFA Score Components, as per Vincent et al, 1996 **eTable 3.** Other Patient Characteristics **eTable 4.** Characterization of Variable Missingness Across the 5 EHR Data Sets **eTable 5.** Spo_2_ Variability for All Spo_2_ Values Within the 5 Minutes Preceding the ABG Measurement **eTable 6.** Percentage of Encounters With Arterial Blood Gases, by Race and Ethnicity **eTable 7.** Rate of ABG Measurements Obtained Throughout a Hospital Encounter by Race and Ethnicity, Stratified by Cardiovascular SOFA Score **eTable 8.** Descriptive Statistics for All Sao_2_-Spo_2_ Pairs With Spo_2 _of at least 88% **eTable 9.** Total Number of Patients per Spo_2_ Level and Characterization of Hidden Hypoxemia Incidence, Stratified by Race and Ethnicity **eTable 10.** Characterization of the Distribution of Hidden Hypoxemia by Respiratory SOFA Score **eTable 11.** Distribution of In-Hospital Mortality, Stratified by Respiratory SOFA Score and Presence of Hidden Hypoxemia **eTable 12.** Descriptive Statistics for Patients With Hidden Hypoxemia vs Patients Without Hypoxemia, Carboxyhemoglobin Less than 2 and Methemoglobin Less Than 2
