# Incorrect Numbers in eTable and Text

**DOI:** 10.1001/jamanetworkopen.2022.1210

**Published:** 2022-02-09

In the Original Investigation titled “Analysis of Discrepancies Between Pulse Oximetry and Arterial Oxygen Saturation Measurements by Race and Ethnicity and Association With Organ Dysfunction and Mortality,”^1^ published November 3, 2021, numerical errors were included in eTable 9 due to author reliance on an older version of this table. This table reports the total number of patients per pulse oximetry level and characterization of hidden hypoxemia incidence, stratified by race and ethnicity. These numbers have been corrected. In addition, the correct numbers for hidden hypoxemia observed in subgroups as reported in the Abstract and Results are “(Black: 1789 [6.9%]; Hispanic: 145 [6.0%]; Asian: 94 [4.9%]; White: 2831 [4.9%]).” The corresponding author has explained these errors in a Comment,^2^ and this article has been corrected.^1^

## References

[1] Wong AKI, Charpignon M, Kim H, . Analysis of discrepancies between pulse oximetry and arterial oxygen saturation measurements by race and ethnicity and association with organ dysfunction and mortality. JAMA Netw Open. 2021;4(11):e2131674. doi:10.1001/jamanetworkopen.2021.31674 34730820 PMC9178439

[2] Wong AKI. Revised discrepancies, MetHb + COHb. Comment. *JAMA Network Open*. January 6, 2022. Accessed January 19, 2022. https://jamanetwork.com/journals/jamanetworkopen/fullarticle/2785794

