# Supplementary material for: Analysis of Discrepancies Between Pulse Oximetry and Arterial Oxygen Saturation Measurements by Race and Ethnicity and Association With Organ Dysfunction and Mortality
Source: JAMA Netw Open. 2021 Nov 3;4(11):e2131674. doi: 10.1001/jamanetworkopen.2021.31674 (PMC9178439; doi:10.1001/jamanetworkopen.2021.31674)
Supplement: Supplement. — eAppendix 1. Supplementary Methods eAppendix 2. Supplementary Results eFigure 1. Spo2-Sao2 by Race and Ethnicity, for All ABG Measurements Taken Throughout 268 904 Hospital Encounters eFigure 2. Spo2-Sao2 by Race and Ethnicity, for the First ABG Measurement per 87 971 Hospital Encounters eFigure 3. Spo2-Sao2 by Race and Ethnicity, for the First ABG Measurement per 87 971 Hospital Encounters, Stratified by Age Group eFigure 4. Spo2-Sao2 by Race and Ethnicity, for the First ABG Measurement per 87 971 Hospital Encounters, Stratified by Sex eFigure 5. Spo2-Sao2 by Race and Ethnicity, for the First ABG Measurement per 87 971 Hospital Encounter, Stratified by Cardiovascular SOFA Score eFigure 6. Directed Acyclic Graph for Race and Ethnicity, Hidden Hypoxemia, Organ Dysfunction, and Mortality eTable 1. Literature Review eTable 2. SOFA Score Components, as per Vincent et al, 1996 eTable 3. Other Patient Characteristics eTable 4. Characterization of Variable Missingness Across the 5 EHR Data Sets eTable 5. Spo2 Variability for All Spo2 Values Within the 5 Minutes Preceding the ABG Measurement eTable 6. Percentage of Encounters With Arterial Blood Gases, by Race and Ethnicity eTable 7. Rate of ABG Measurements Obtained Throughout a Hospital Encounter by Race and Ethnicity, Stratified by Cardiovascular SOFA Score eTable 8. Descriptive Statistics for All Sao2-Spo2 Pairs With Spo2 of at least 88% eTable 9. Total Number of Patients per Spo2 Level and Characterization of Hidden Hypoxemia Incidence, Stratified by Race and Ethnicity eTable 10. Characterization of the Distribution of Hidden Hypoxemia by Respiratory SOFA Score eTable 11. Distribution of In-Hospital Mortality, Stratified by Respiratory SOFA Score and Presence of Hidden Hypoxemia eTable 12. Descriptive Statistics for Patients With Hidden Hypoxemia vs Patients Without Hypoxemia, Carboxyhemoglobin Less than 2 and Methemoglobin Less Than 2 [file jamanetwopen-e2131674-s001.pdf]

Supplemental Online Content

Wong AKI, Charpignon M, Kim H, et al. Analysis of discrepancies between pulse oximetry and arterial oxygen saturation measurements by race and ethnicity and association with organ dysfunction and mortality. *JAMA Netw Open.* 2021;4(11):e2131674. doi:10.1001/jamanetworkopen.2021.31674

eAppendix 1. Supplementary Methods

eAppendix 2. Supplementary Results

eFigure 1. SpO_2-SaO_2 by Race and Ethnicity, for All ABG Measurements Taken Throughout 268 904 Hospital Encounters

eFigure 2. SpO_2-SaO_2 by Race and Ethnicity, for the First ABG Measurement per 87 971 Hospital Encounters

eFigure 3. SpO_2-SaO_2 by Race and Ethnicity, for the First ABG Measurement per 87 971 Hospital Encounters, Stratified by Age Group

eFigure 4. SpO_2-SaO_2 by Race and Ethnicity, for the First ABG Measurement per 87 971 Hospital Encounters, Stratified by Sex

eFigure 5. SpO_2-SaO_2 by Race and Ethnicity, for the First ABG Measurement per 87 971 Hospital Encounter, Stratified by Cardiovascular SOFA Score

eFigure 6. Directed Acyclic Graph for Race and Ethnicity, Hidden Hypoxemia, Organ Dysfunction, and Mortality

eTable 1. Literature Review

eTable 2. SOFA Score Components, as per Vincent et al, 1996

eTable 3. Other Patient Characteristics

eTable 4. Characterization of Variable Missingness Across the 5 EHR Data Sets

eTable 5. SpO_2 Variability for All SpO_2 Values Within the 5 Minutes Preceding the ABG Measurement

eTable 6. Percentage of Encounters With Arterial Blood Gases, by Race and Ethnicity

eTable 7. Rate of ABG Measurements Obtained Throughout a Hospital Encounter by Race and Ethnicity, Stratified by Cardiovascular SOFA Score

eTable 8. Descriptive Statistics for All SaO_2-SpO_2 Pairs With SpO_2 of at Least 88%

eTable 9. Total Number of Patients per SpO_2 Level and Characterization of Hidden Hypoxemia Incidence, Stratified by Race and Ethnicity

eTable 10. Characterization of the Distribution of Hidden Hypoxemia by Respiratory SOFA Score

eTable 11. Distribution of In-Hospital Mortality, Stratified by Respiratory SOFA Score and Presence of Hidden Hypoxemia

eTable 12. Descriptive Statistics for Patients With Hidden Hypoxemia vs Patients Without Hypoxemia, Carboxyhemoglobin Less than 2 and Methemoglobin Less Than 2

This supplemental material has been provided by the authors to give readers additional information about their work.

© 2021 Wong AI et al. *JAMA Network Open.*

eAppendix 1. Supplementary Methods

SpO_2 variability over five minutes

To further characterize SpO_2 variability before an ABG, all SpO_2 assessments were evaluated in the five minutes before the ABG. SpO_2 variability was assessed by the difference between the maximum and the minimum values recorded during this time period.

Co-oximetry

98.2% of all ABGs in Emory Healthcare had co-oximetry, identifying MetHb and COHb.

rSOFA and hidden hypoxemia

Potential association between hidden hypoxemia and rSOFA, was closely examined. First, the rate of hidden hypoxemia was stratified by rSOFA score to determine whether risk for hidden hypoxemia increased with higher rSOFA. Second, in-hospital mortality was stratified by both rSOFA score and hidden hypoxemia to determine whether hidden hypoxemia ($SpO2 \geq 88\%$ despite $SaO2 < 88\%$) independently increased in-hospital mortality after adjustment from rSOFA.

Multivariate modeling

Multivariate models were examined both without and with laboratory values.

- Set without labs: SpO_2, age, sex, BMI, race/ethnicity, organ dysfunction scores @ t_{ABG} (SOFA score, RSOFA, CVSOFA)
- Set with labs: SpO_2, age, sex, BMI, race/ethnicity, organ dysfunction scores @ t_{ABG} (SOFA score, RSOFA, CVSOFA), laboratory values @ t_{ABG} (serum lactate, serum creatinine)

< The remainder of this page intentionally left blank. >

eAppendix 2. Supplementary Results

SpO_2 variability over five minutes

From 653,042 SpO_2-SaO_2 pairs across all encounters and all ABGs, with SpO_2 recorded within five minutes prior to an ABG. SpO_2 had an average variation of 0.21% ± a standard deviation of 0.70% per SaO_2 value.

© 2021 Wong AI et al. *JAMA Network Open.*

eFigure 1. SpO2-SaO2 by Race and Ethnicity, for All ABG Measurements Taken Throughout 268 904 Hospital Encounters

Boxplot of the SpO_2 (x-axis) vs. SaO_2 (y-axis) values for each encounter, with White patients as the reference standard in blue on the right side of each paired dataset. The blue diagonal dotted line represents the 1:1 SaO_2-SpO_2 correlation line. The red dashed line indicates SaO_2=88%.

eFigure 1a. Distribution of SaO_2 per SpO_2 level by race-ethnicity for Asian (n=1,919) compared with White (n=57,623) patients

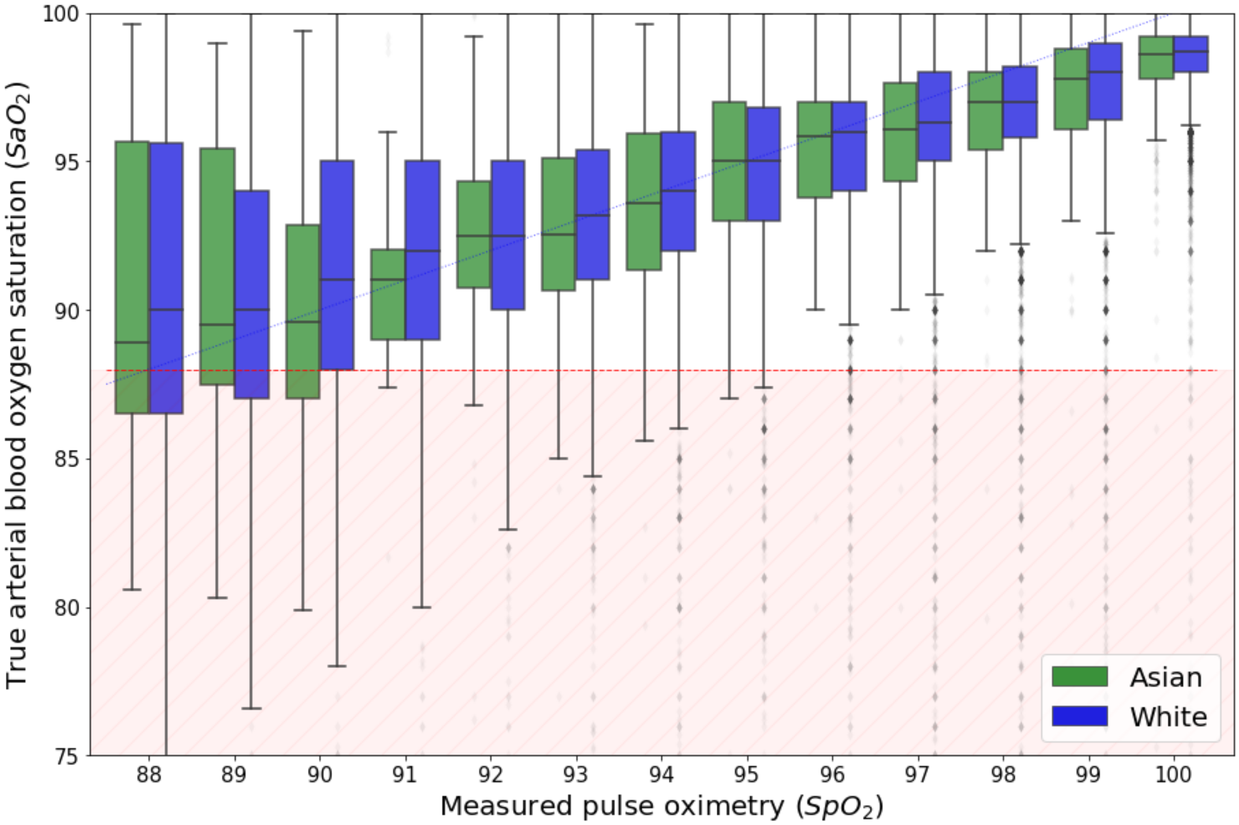

© 2021 Wong AI et al. *JAMA Network Open.*

eFigure 1b. Distribution of SaO_2 per SpO_2 level by race-ethnicity for Black (n=26,032) compared with White (n=57,623) patients

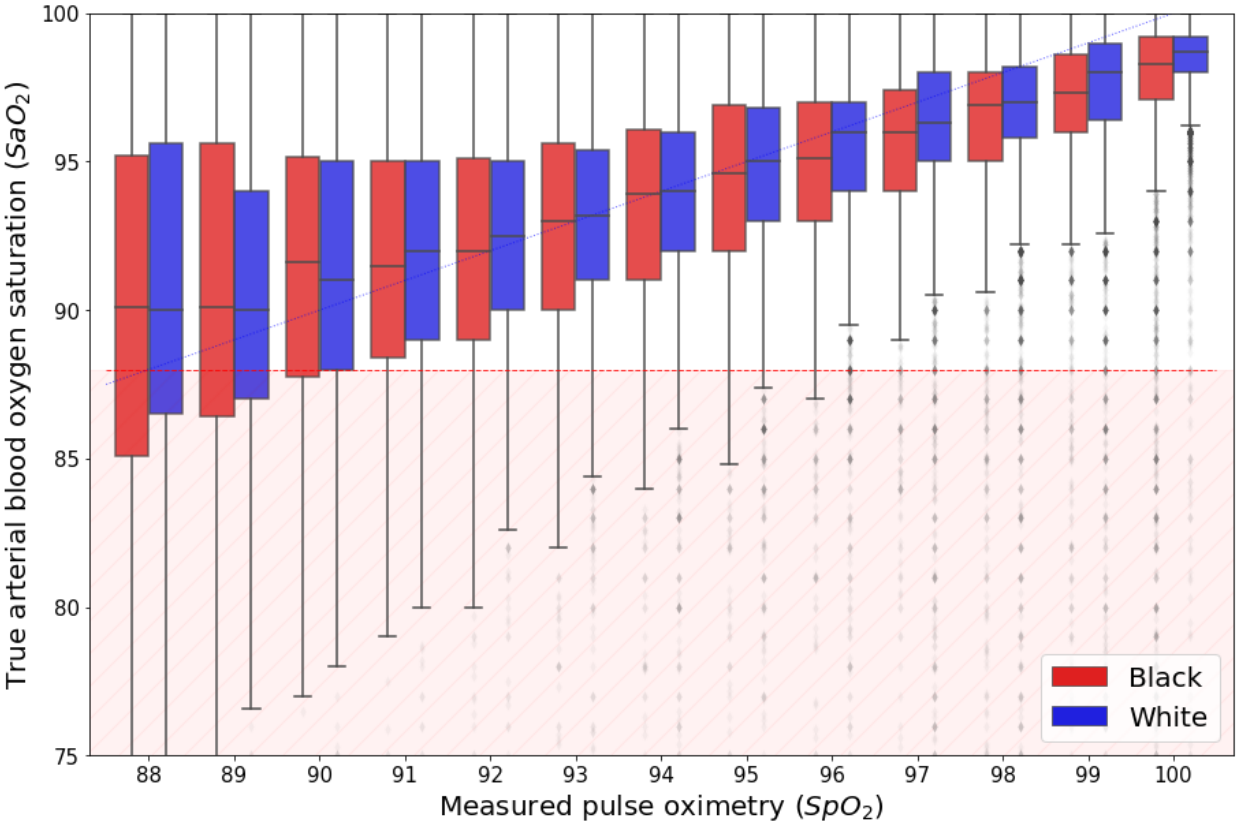

© 2021 Wong AI et al. *JAMA Network Open.*

eFigure 1c. Distribution of SaO_2 per SpO_2 level by race-ethnicity for Hispanic (n=2,397) compared with White (n=57,623) patients

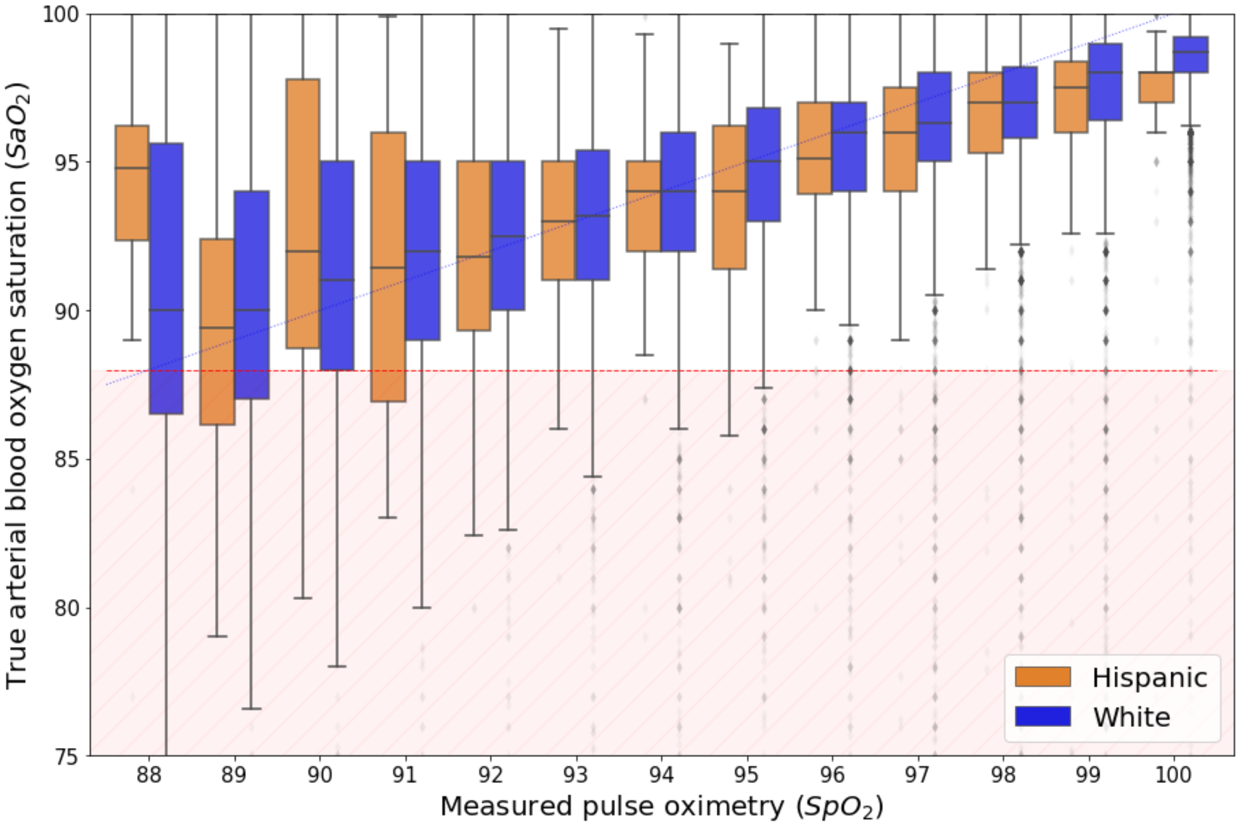

© 2021 Wong AI et al. *JAMA Network Open.*

eFigure 2. SpO_2-SaO_2 by Race and Ethnicity, for the First ABG Measurement per 87 971 Hospital Encounters

In this figure, the closest SpO_2 value for all ABGs during a hospitalization that had an SpO_2 measured within five minutes preceding an ABG. Results are presented for SpO_2 values ranging from 88% to 100%. This differs from Figure 2 by scale (268,904 vs. 87,971), and is potentially affected by repeated measures. Each of the three subfigures includes a pairwise comparison by race-ethnicity: Asian, Black, or Hispanic compared with White (selected as the reference, because White has the highest prevalence in the dataset).

eFigure 2a. Distribution of SaO_2 per SpO_2 level for Asian (n=6,005) compared with White (n=186,170) patients

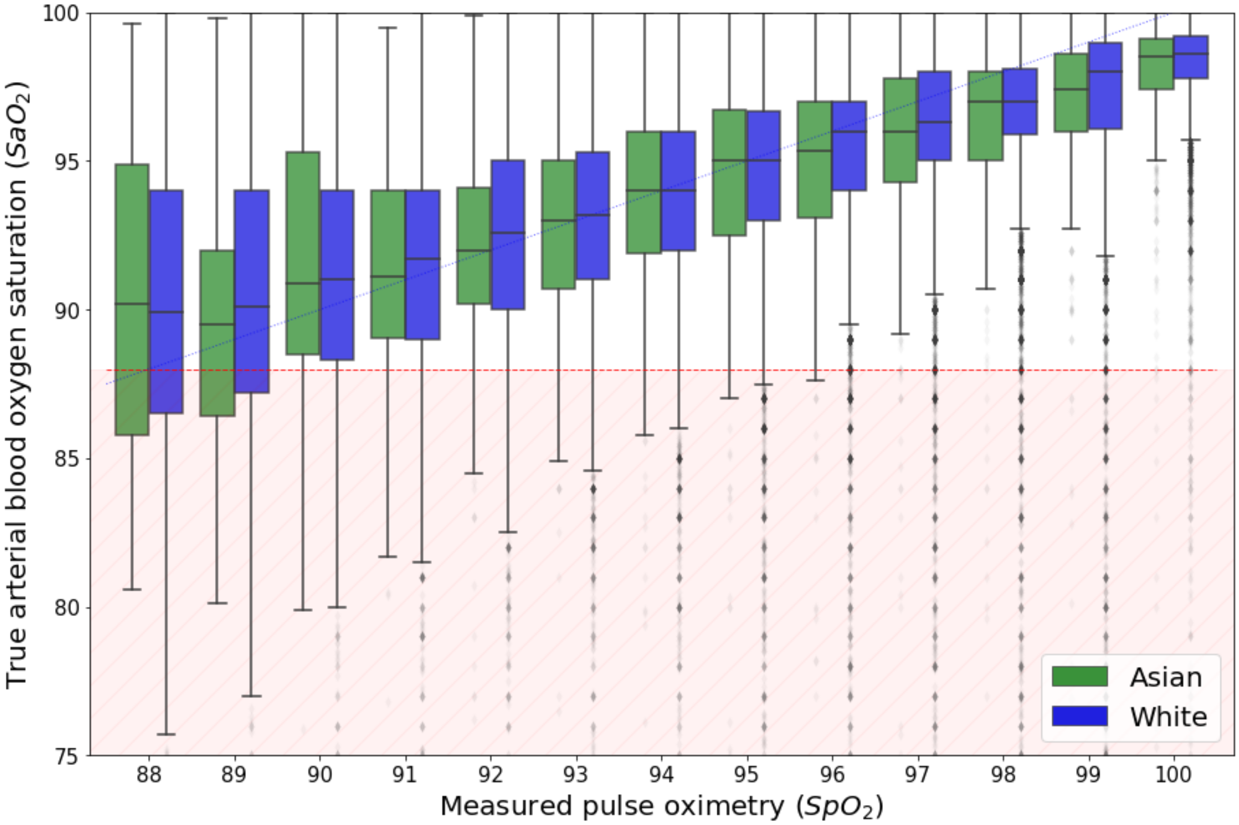

© 2021 Wong AI et al. *JAMA Network Open.*

eFigure 2b. Distribution of SaO2 per SpO2 level for Black (n=67,724) compared with White (n=186,170) patients

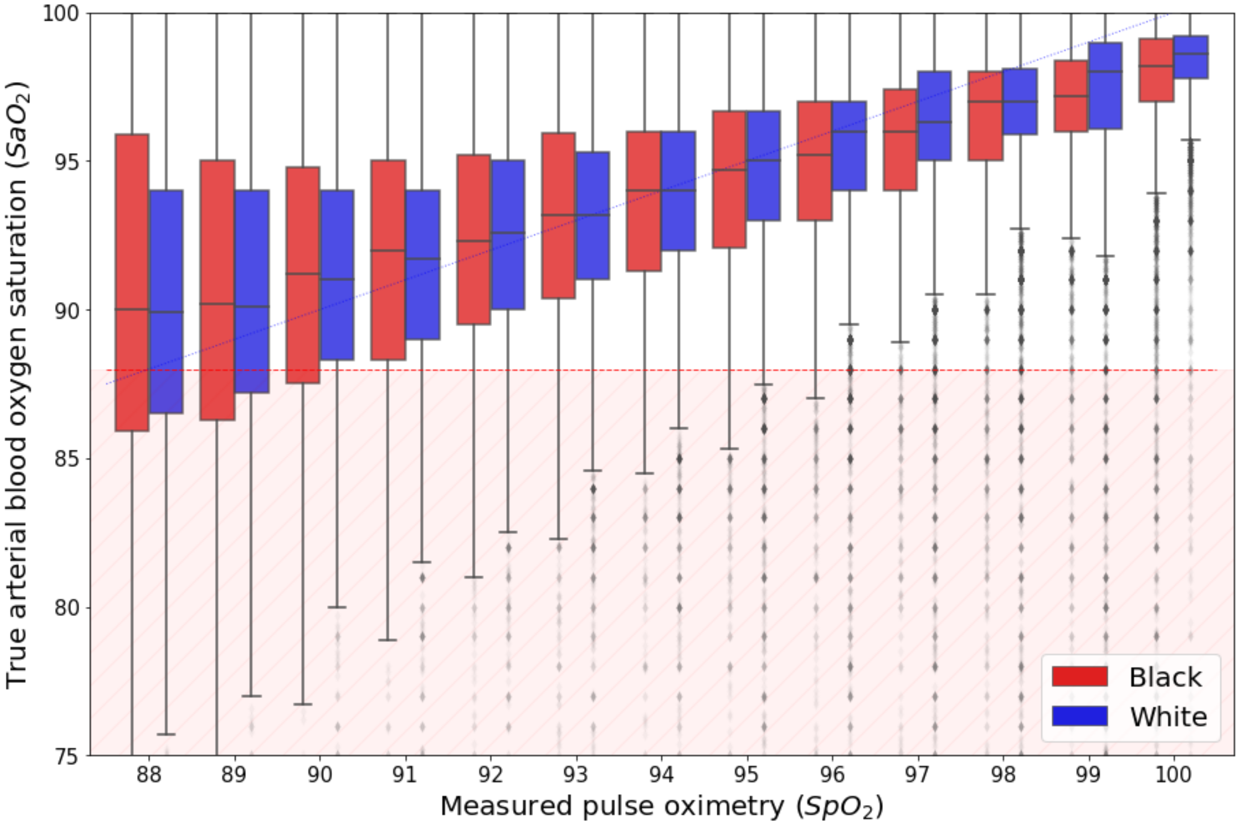

© 2021 Wong AI et al. *JAMA Network Open.*

eFigure 2c. Distribution of SaO_2 per SpO_2 level for Hispanic (n=9,005) compared with White (n=186,170) patients

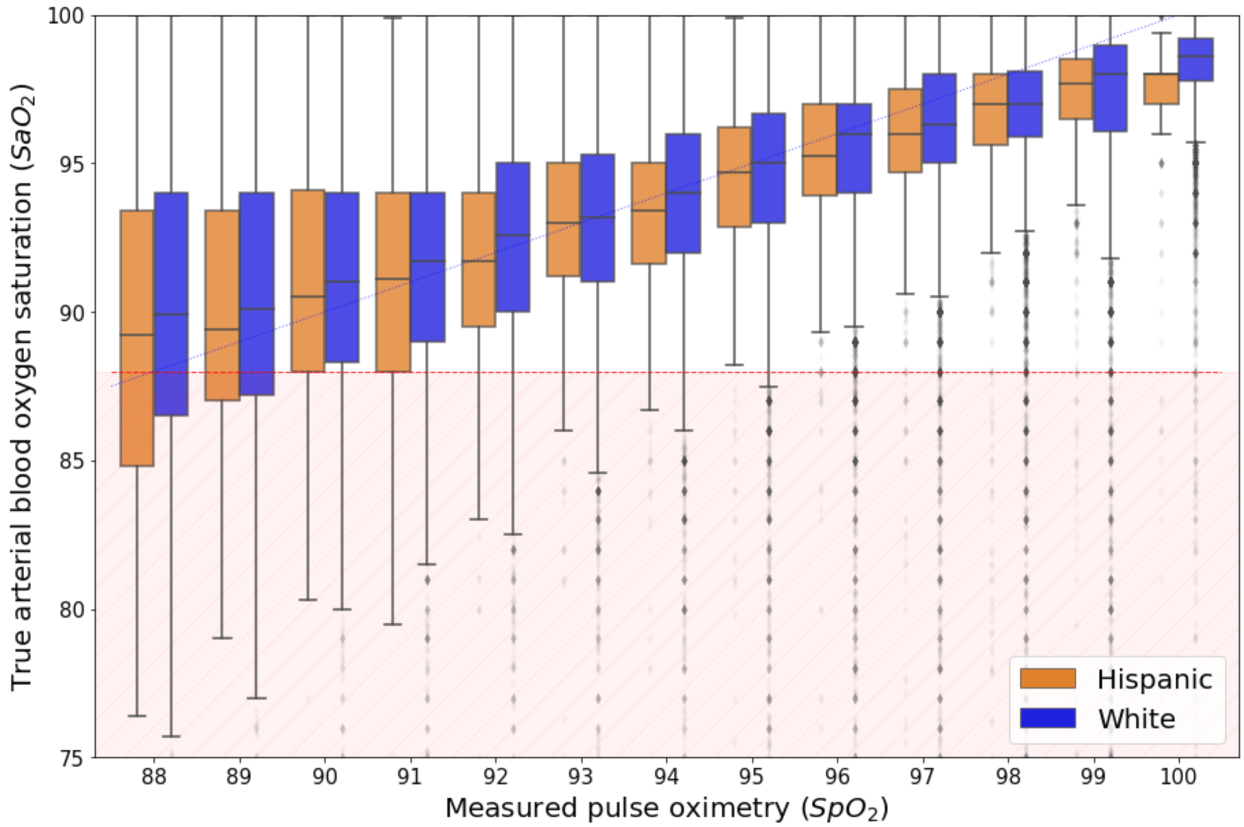

© 2021 Wong AI et al. *JAMA Network Open.*

eFigure 3. SpO_2-SaO_2 by Race and Ethnicity, for the First ABG Measurement per 87 971 Hospital Encounters, Stratified by Age Group

Box plots of the SpO_2 (x-axis) vs. SaO_2 (y-axis) values are presented by race-ethnicity for the first ABG per hospital encounter, stratified by age group (age<65, age≥65).

Left panels feature patients <65 years old. Right panels feature patients ≥65 years old.

eFigure 3a. Distribution of SaO_2 per SpO_2 level for Asian (n=1,919) compared with White (n=57,623) patients, stratified by age group

Age<65: Asian (n=948) compared with White (n=26,317)

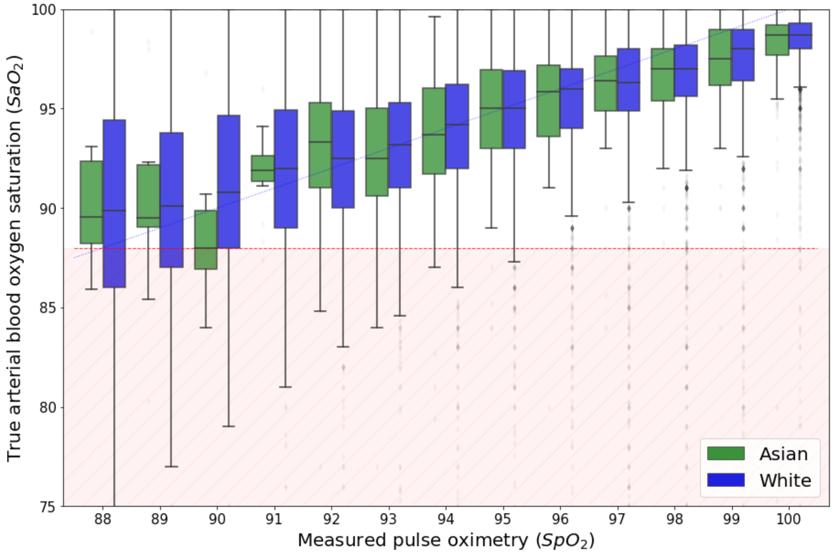

Age≥ 65: Asian (n=971) compared with White (n=31,306)

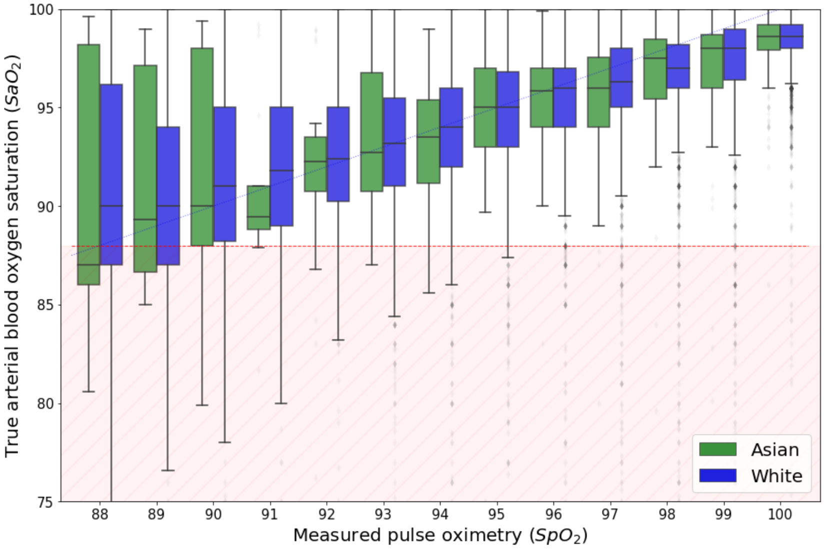

© 2021 Wong AI et al. *JAMA Network Open.*

eFigure 3b. Distribution of SaO_2 per SpO_2 level for Black (n=26,032) compared with White (n=57,623) patients, stratified by age group

Age<65: Black (n=16,419) compared with White (n=26,317)

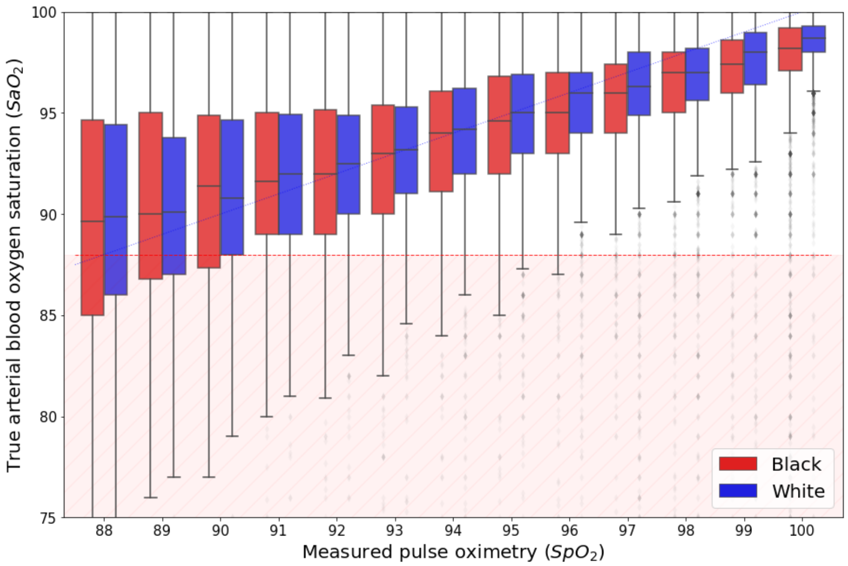

Age≥ 65: Black (n=9,613) compared with White (n=31,306)

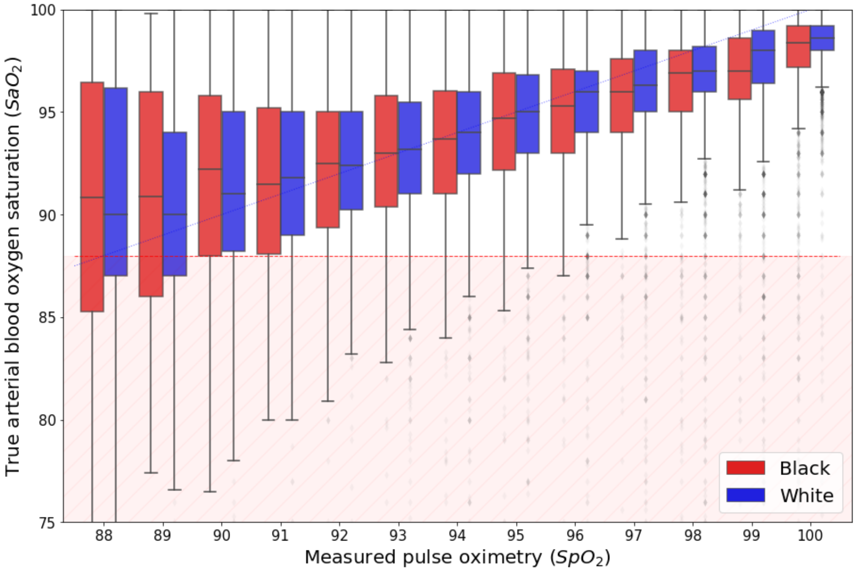

© 2021 Wong AI et al. *JAMA Network Open.*

eFigure 3c. Distribution of SaO_2 per SpO_2 level for Hispanic (n=2,397) compared with White (n=52,623) patients, stratified by age group

Age<65: Hispanic (n=1,302) compared with White (n=26,317)

Age≥ 65: Hispanic (n=1,095) compared with White (n=31,306)

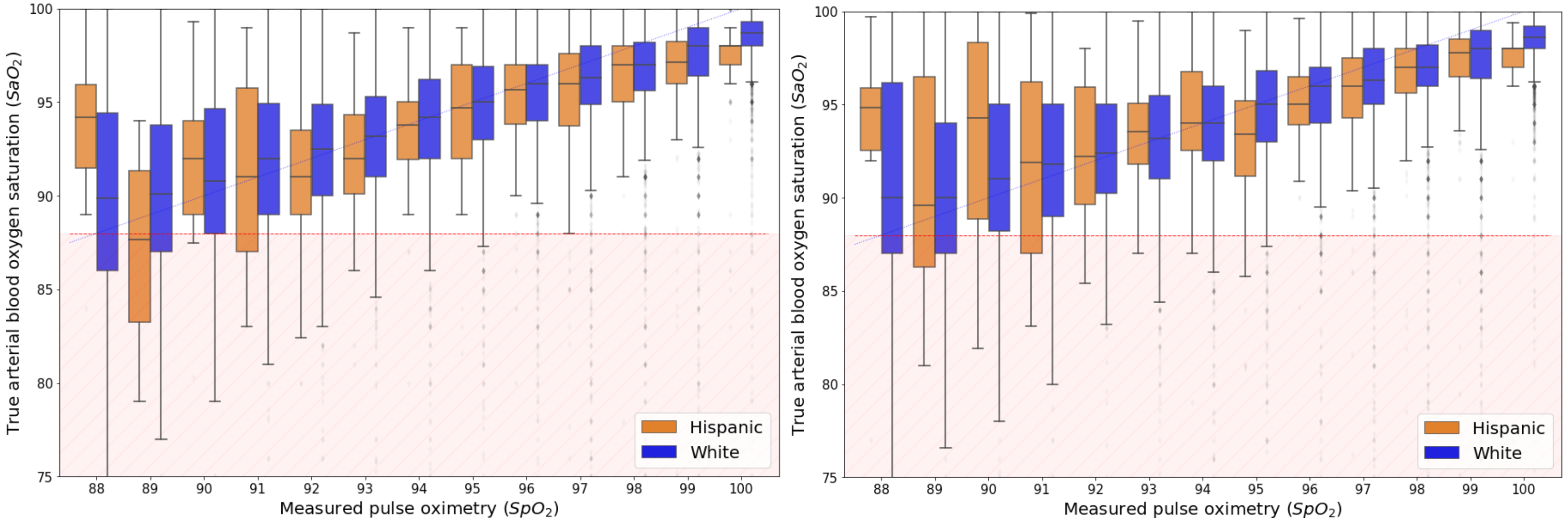

© 2021 Wong AI et al. *JAMA Network Open.*

eFigure 4. SpO_2-SaO_2 by Race and Ethnicity, for the First ABG Measurement per 87 971 Hospital Encounter, Stratified by Sex

Box plots of the SpO_2 (x-axis) vs. SaO_2 (y-axis) values are presented by race-ethnicity for the first ABG per encounter, stratified by sex (male, female).

Left panels feature male patients. Right panels feature female patients.

eFigure 4a. Distribution of SaO_2 per SpO_2 level for Asian (n=1,919) compared with White (n=52,623) patients, stratified by sex

Males: Asian (n=1,156) compared with White (n=33,794)

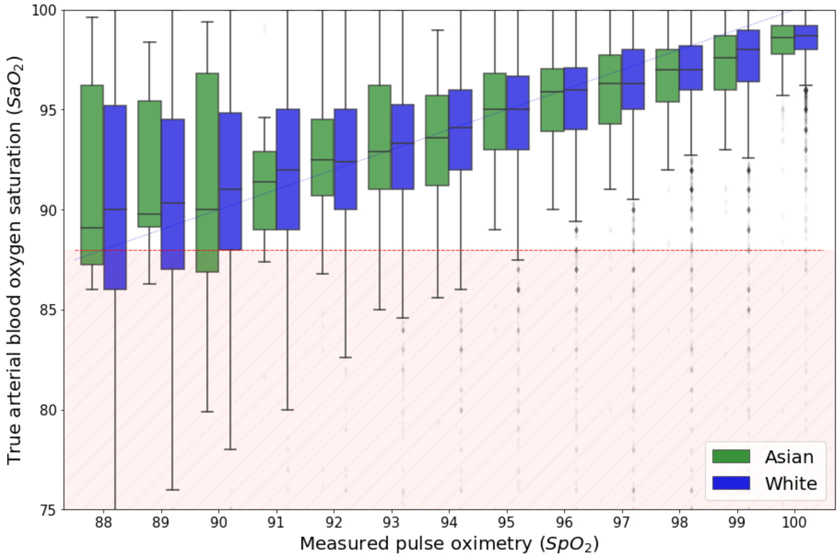

Females: Asian (n=763) compared with White (n=23,829)

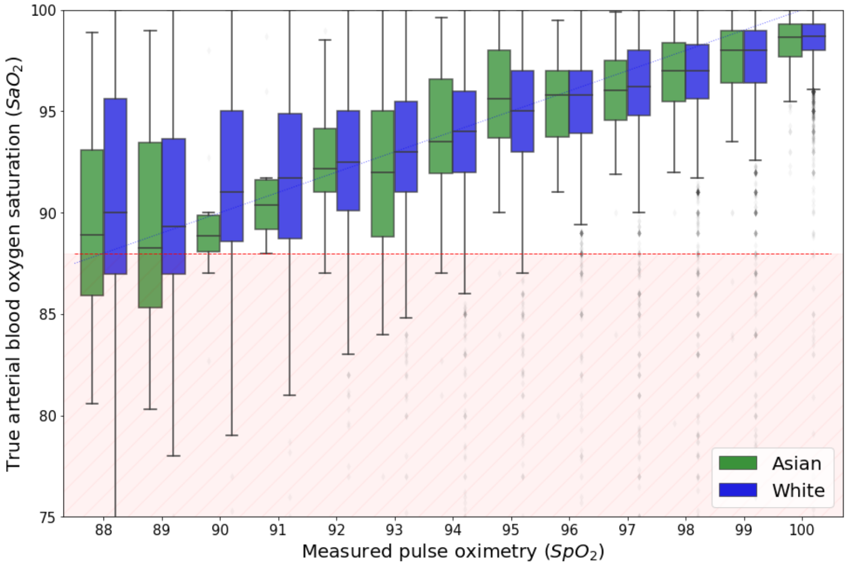

© 2021 Wong AI et al. *JAMA Network Open.*

eFigure 4b. Distribution of SaO_2 per SpO_2 level for Black (n=26,032) compared with White (n=57,723) patients, stratified by sex

Males: Black (n=13,904) compared with White (n=33,794)

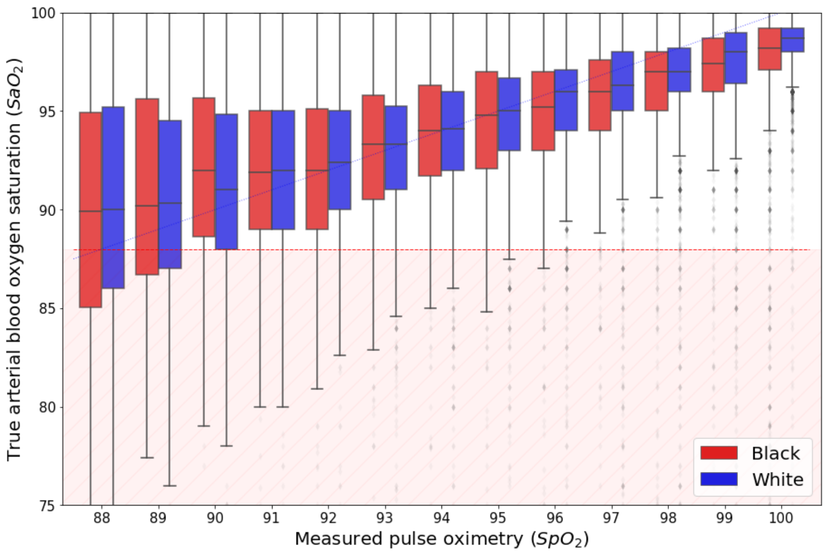

Females: Black (n=12,128) compared with White (n=23,829)

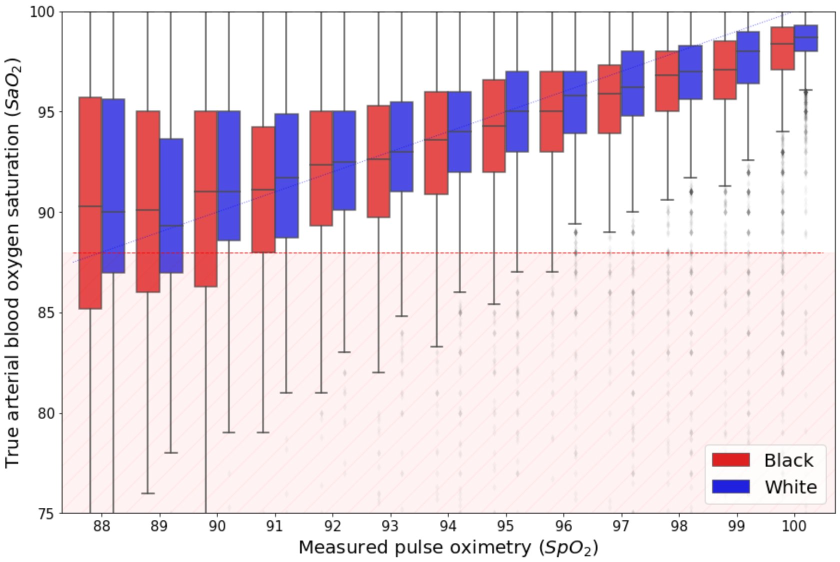

© 2021 Wong AI et al. *JAMA Network Open.*

eFigure 4c. Distribution of SaO_2 per SpO_2 level for Hispanic (n=2,397) compared with White (n=57,623) patients, stratified by sex

Males: Hispanic (n=1,404) compared with White (n=33,794)

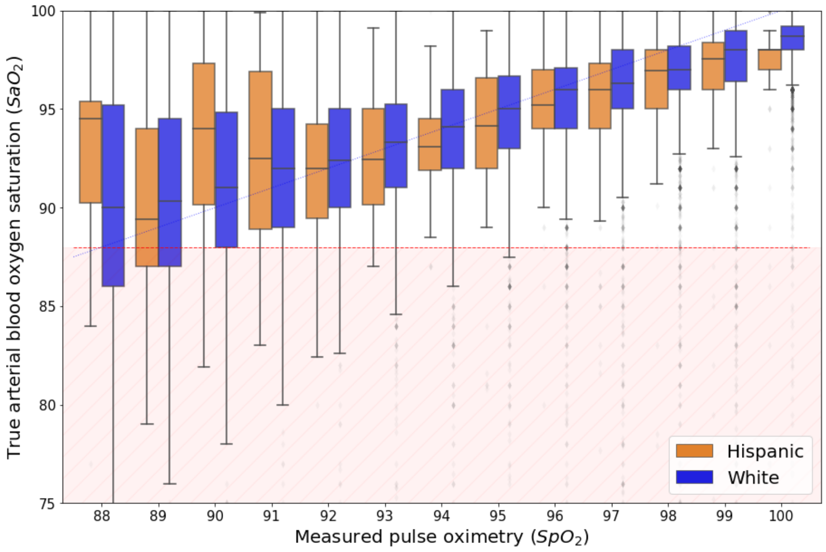

Females: Hispanic (n=993) compared with White (n=23,829)

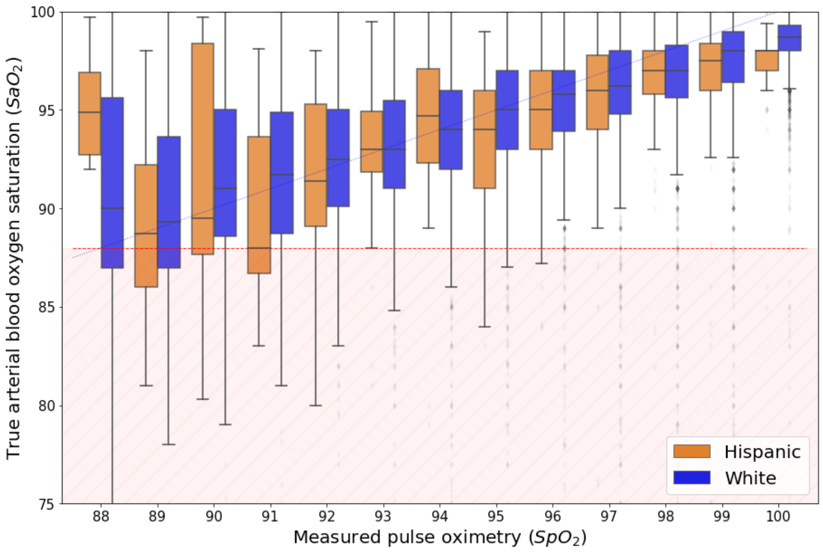

© 2021 Wong AI et al. *JAMA Network Open.*

eFigure 5. SpO_2-SaO_2 by Race and Ethnicity, for the First ABG Measurement per 87 971 Hospital Encounter, Stratified by Cardiovascular SOFA Score

Box plots of the SpO_2 (x-axis) vs. SaO_2 (y-axis) values are presented by race-ethnicity for the first ABG per hospital encounter, stratified by cardiovascular SOFA score.

<The remainder of this page intentionally left blank.>

© 2021 Wong AI et al. *JAMA Network Open.*

eFigure 5a. Distribution of SaO_2 per SpO_2 level for Asian (n=689) compared with White (n=36,681) patients, stratified by cardiovascular SOFA score

cvSOFA = 0: Asian (n=259) compared with White (n=11,645*)*

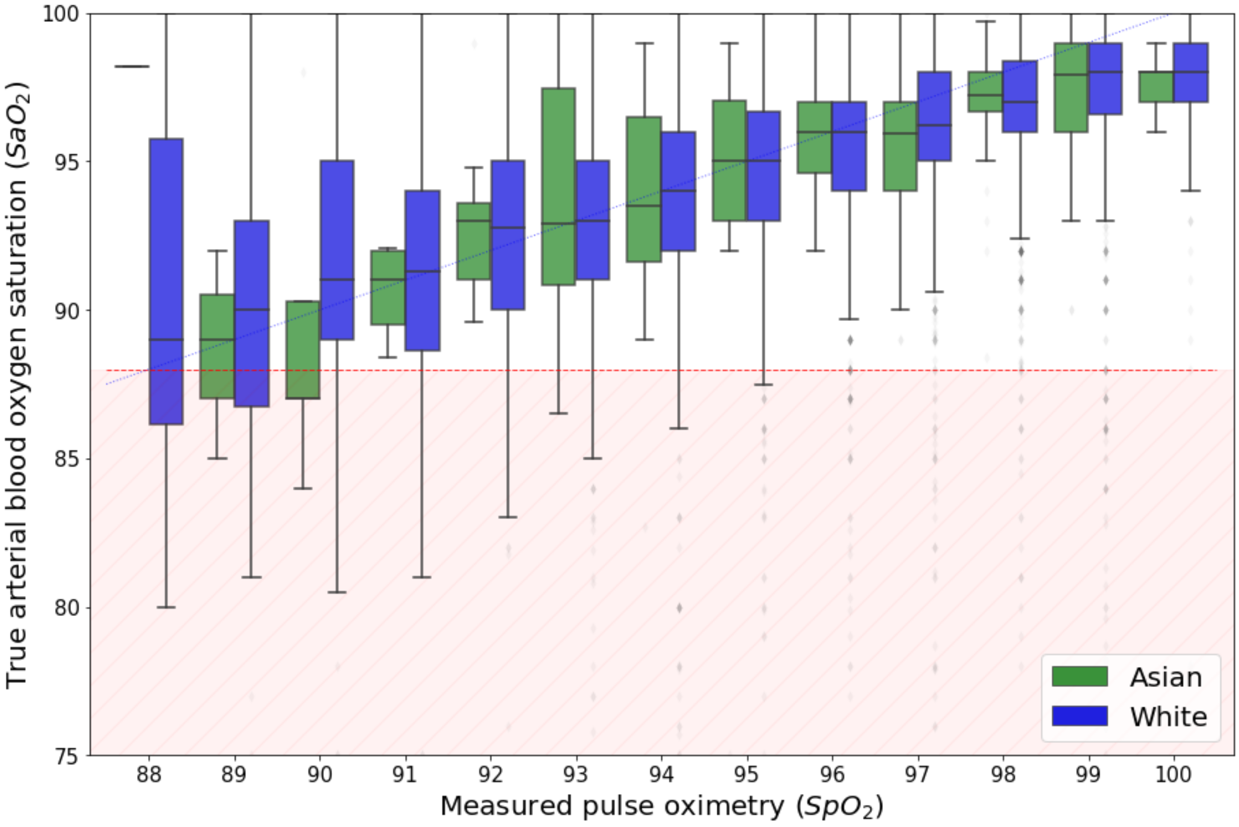

© 2021 Wong AI et al. *JAMA Network Open.*

cvSOFA = 1: Asian (n=388) compared with White (n=18,112)

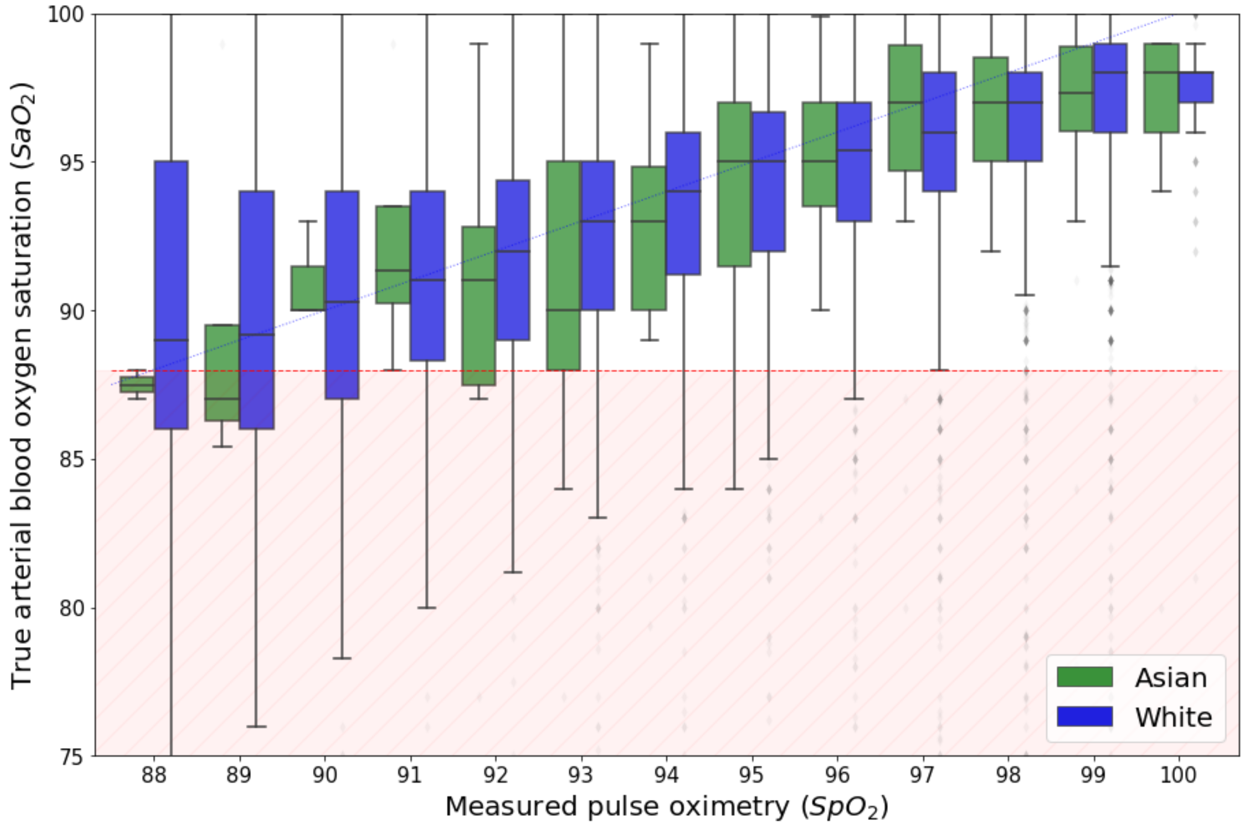

© 2021 Wong AI et al. *JAMA Network Open.*

cvSOFA = 2: Asian (n=9) compared with White (*n=681)*

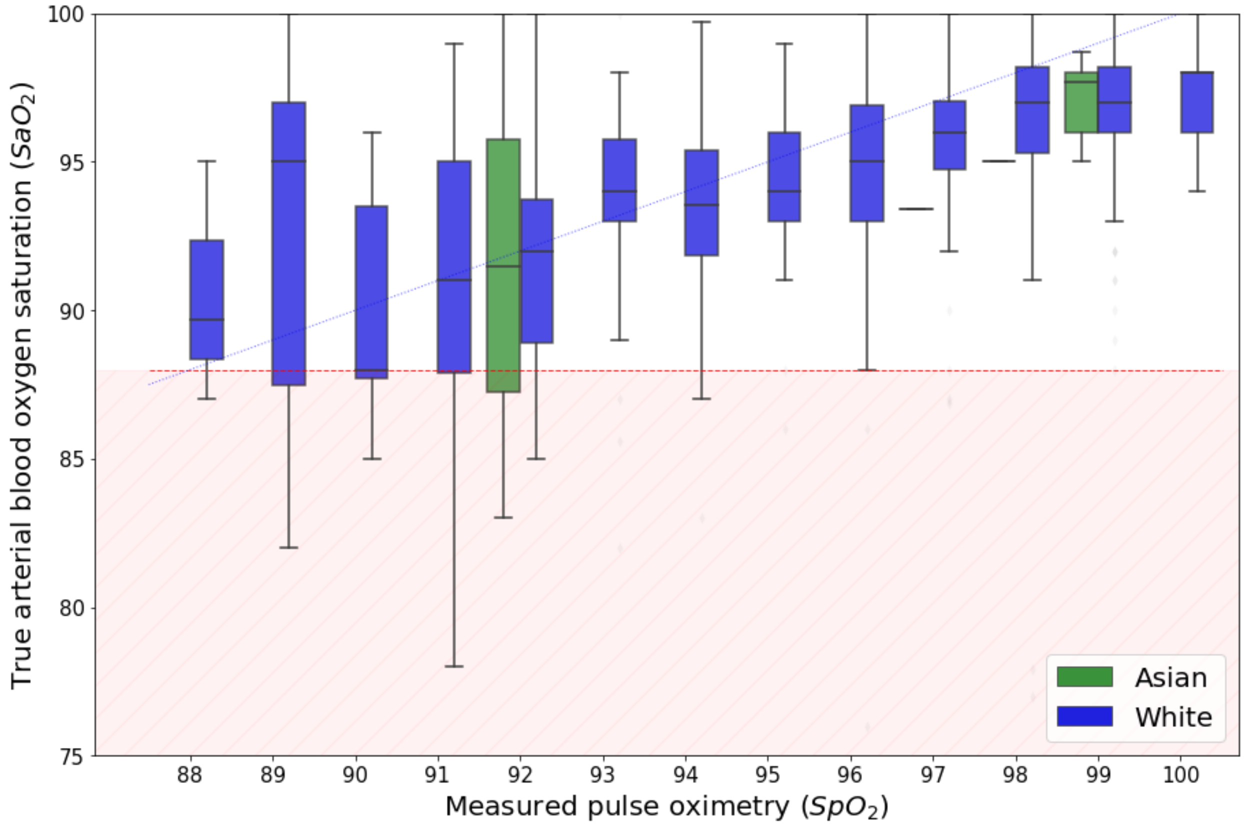

© 2021 Wong AI et al. *JAMA Network Open.*

cvSOFA = 3: *Asian (n*=38) compared with White (n=1,955)

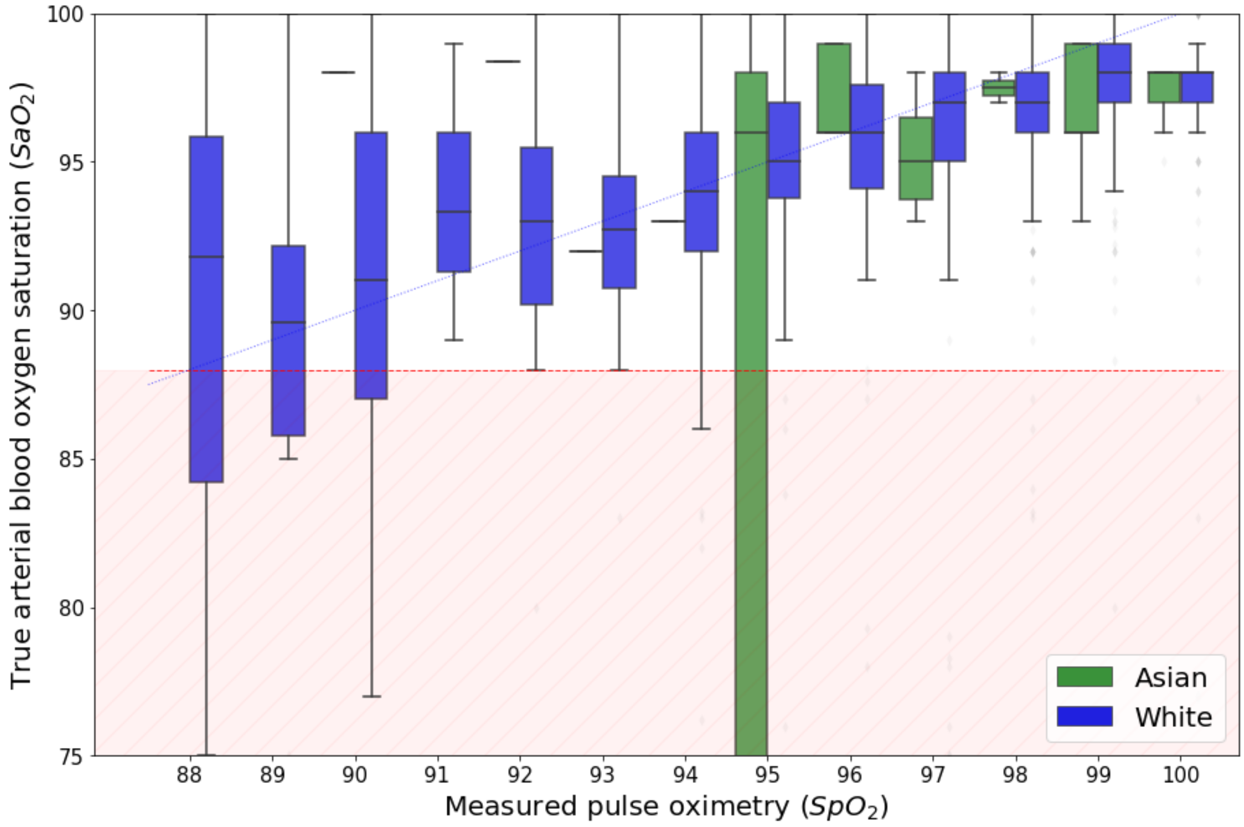

© 2021 Wong AI et al. *JAMA Network Open.*

cvSOFA = 4: Asian (n=31) compared with White (n=610)

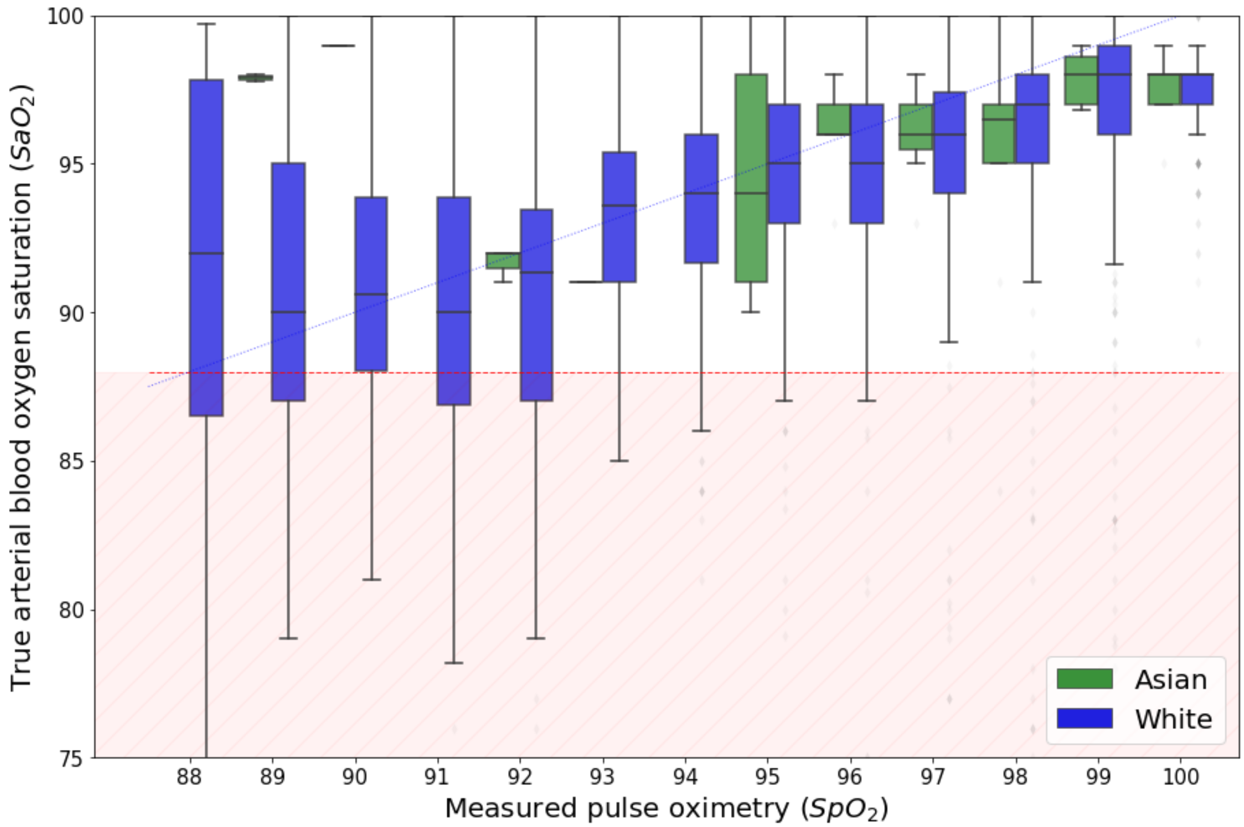

© 2021 Wong AI et al. *JAMA Network Open.*

eFigure 5b. Distribution of SaO_2 per SpO_2 level for Black (n=4,030) compared with White (n=36,681) patients, stratified by cardiovascular SOFA score

cvSOFA = 0: Black (n=1,666) compared with White (n=11,645)

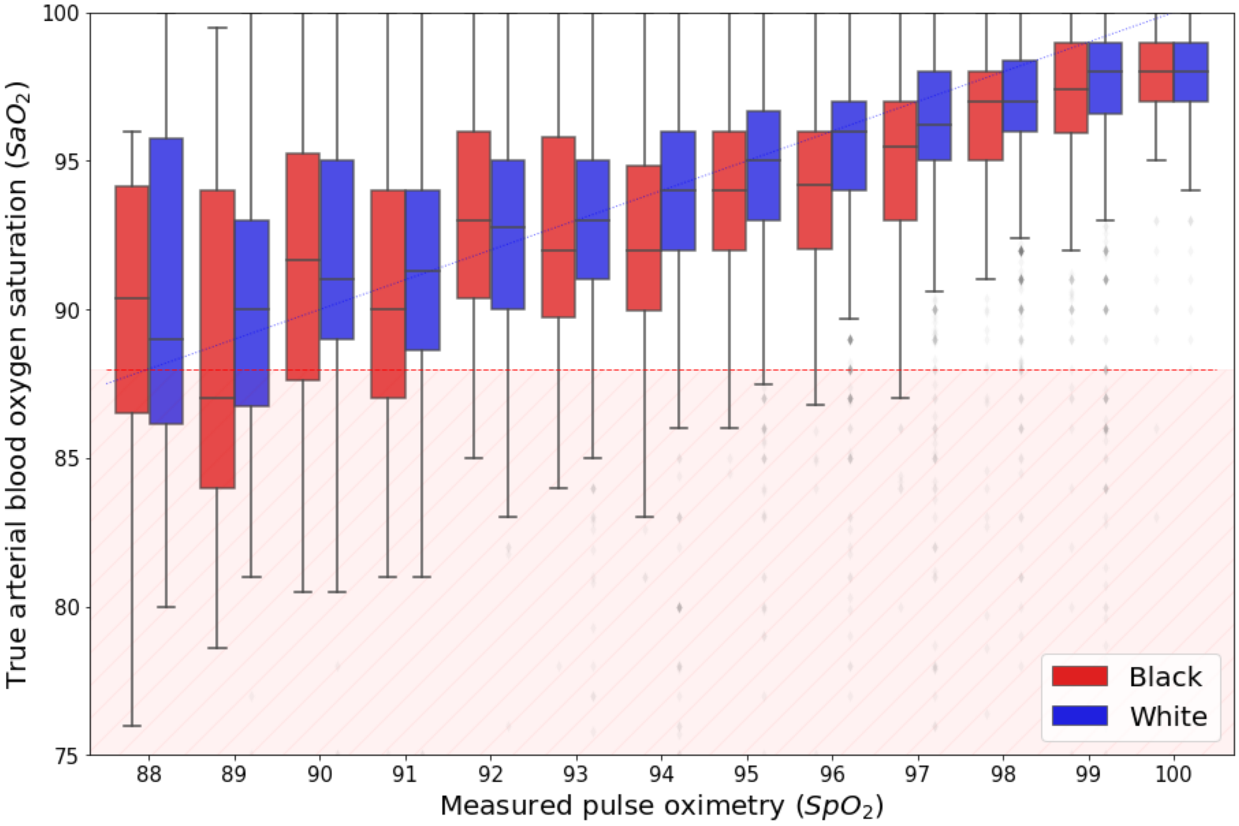

© 2021 Wong AI et al. *JAMA Network Open.*

cvSOFA = 1: Black (n=1,754) compared with White (n=18,112)

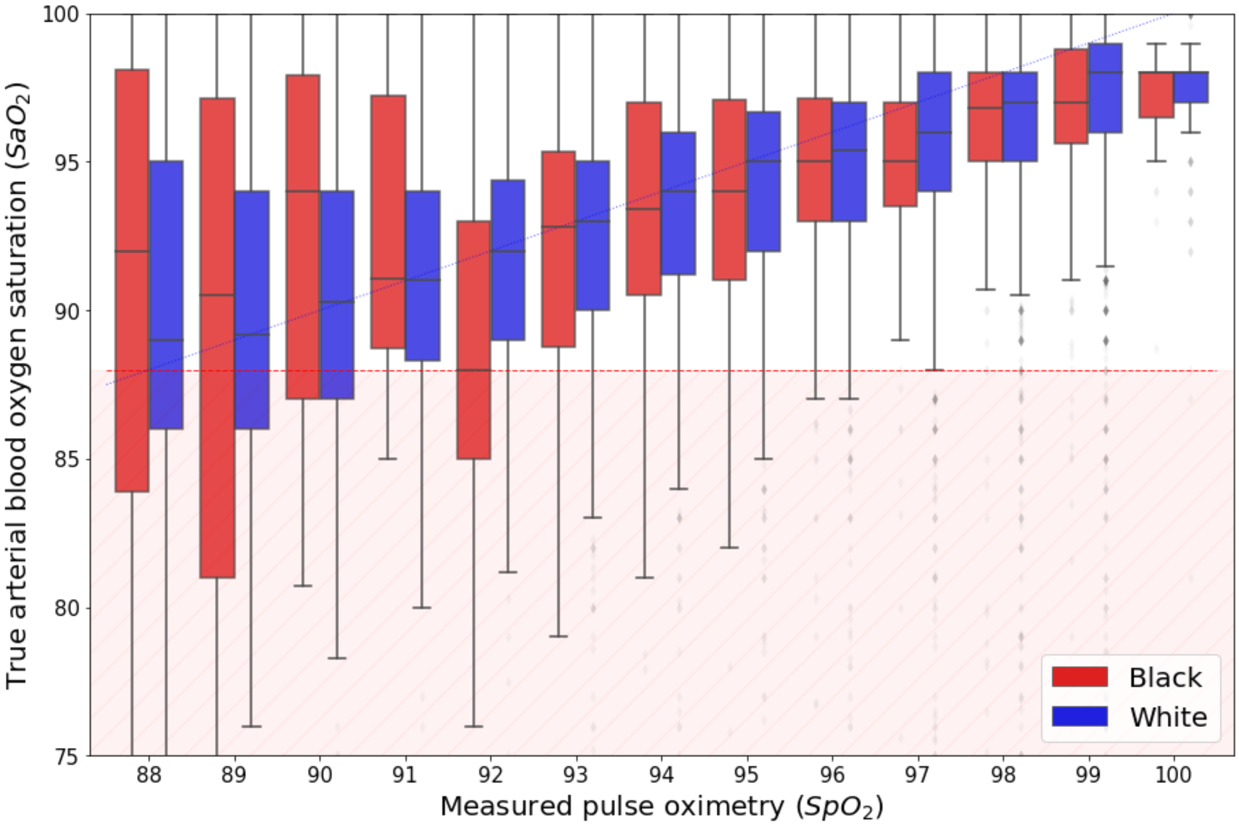

© 2021 Wong AI et al. *JAMA Network Open.*

cvSOFA = 2: Black (n=75) compared with White (681)

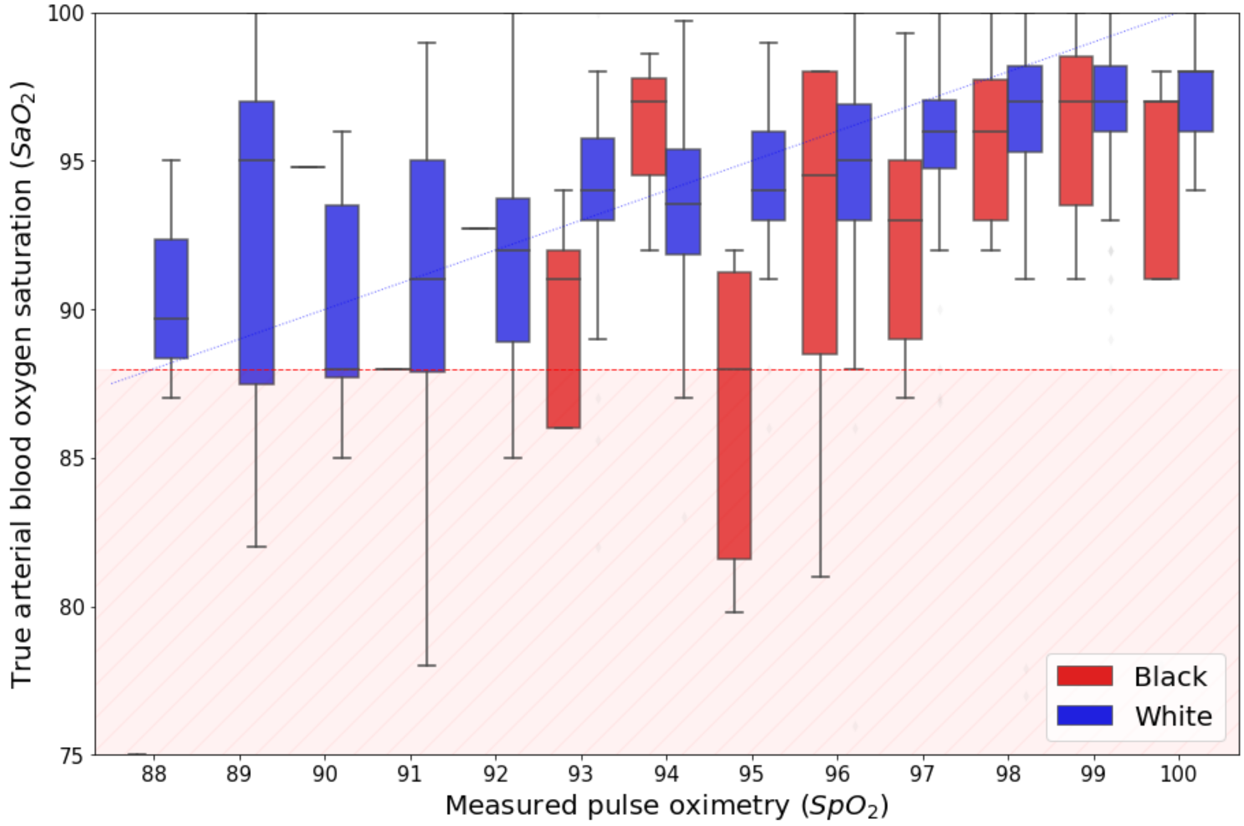

© 2021 Wong AI et al. *JAMA Network Open.*

cvSOFA = 3: Black (n=149) compared with White (n=1,955)

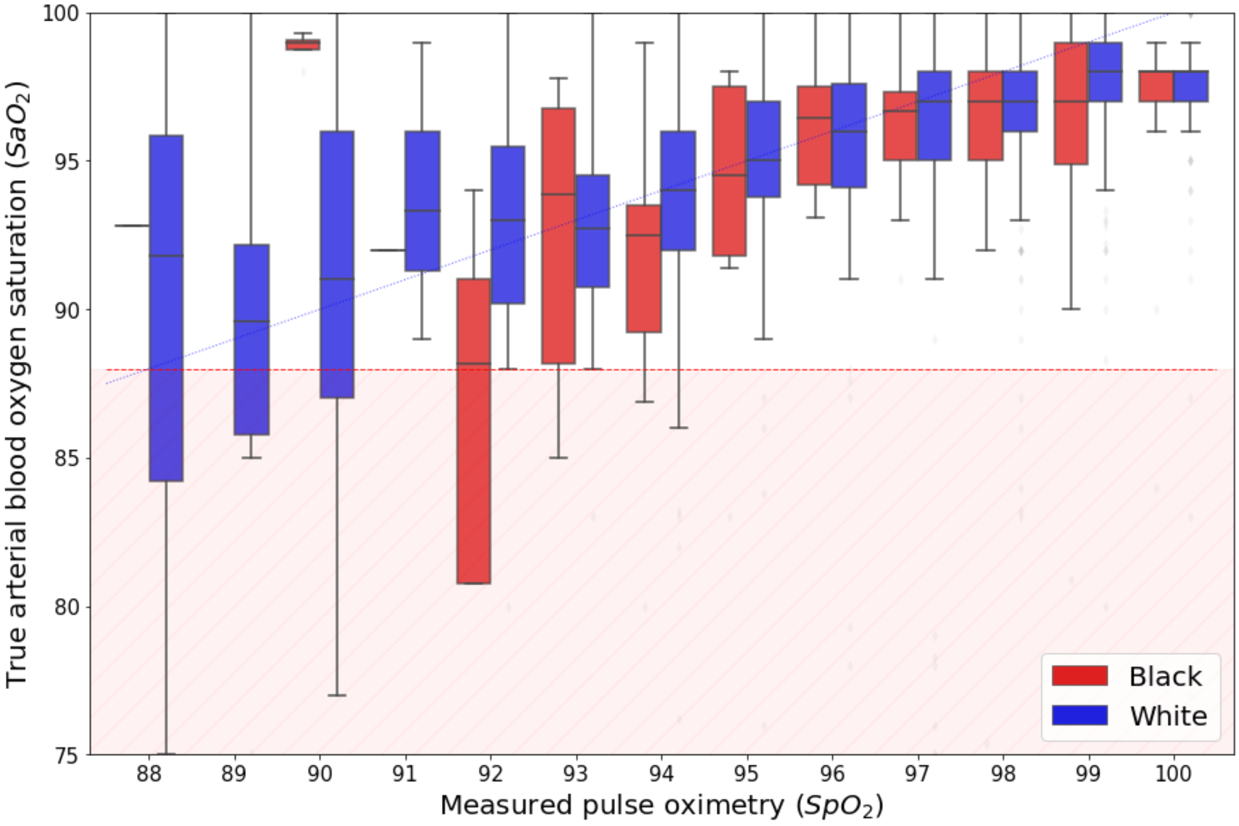

© 2021 Wong AI et al. *JAMA Network Open.*

cvSOFA = 4: Black (n=293) compared with White (n=2,487)

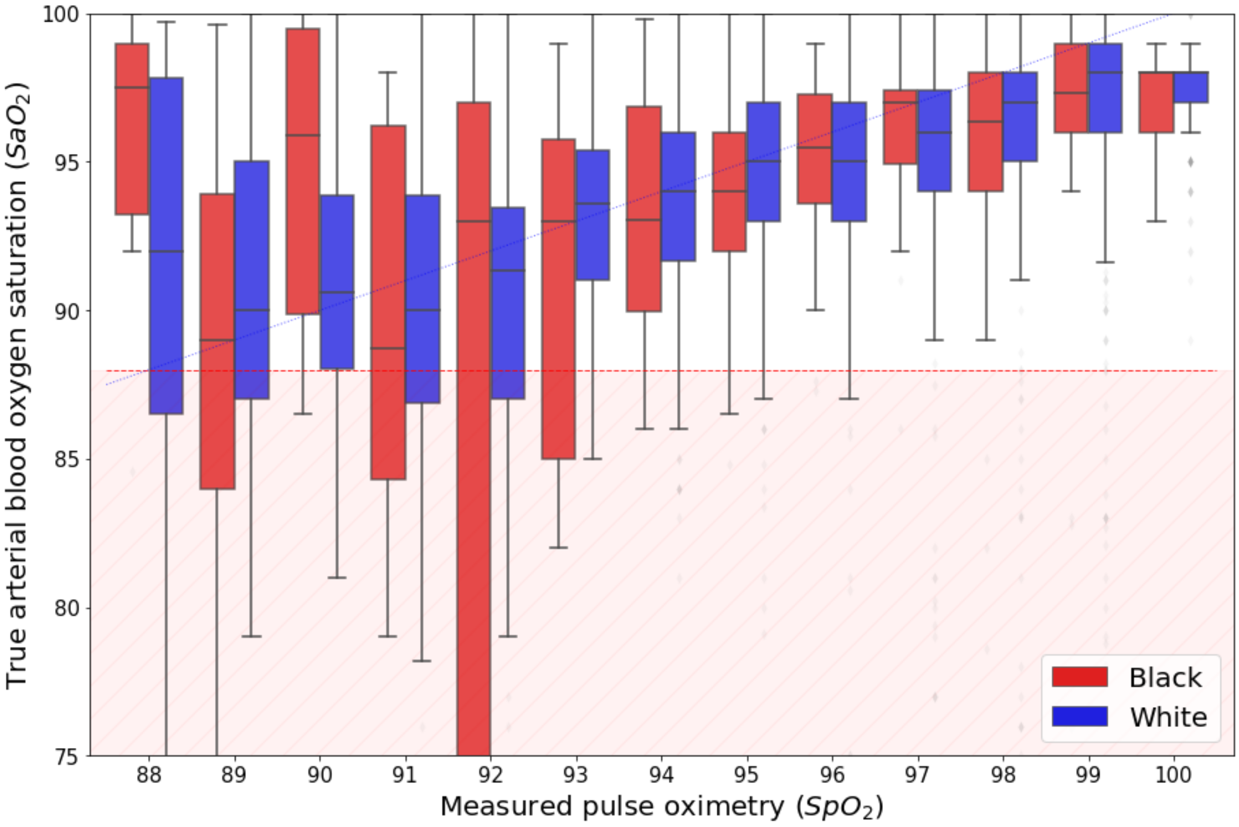

© 2021 Wong AI et al. *JAMA Network Open.*

eFigure 5c. Distribution of SaO2 per SpO2 level for Hispanic (n=1,910) compared with White (n=36,681) patients, stratified by cardiovascular SOFA score

cvSOFA = 0: Hispanic (n=620) compared with White (n=11,645)

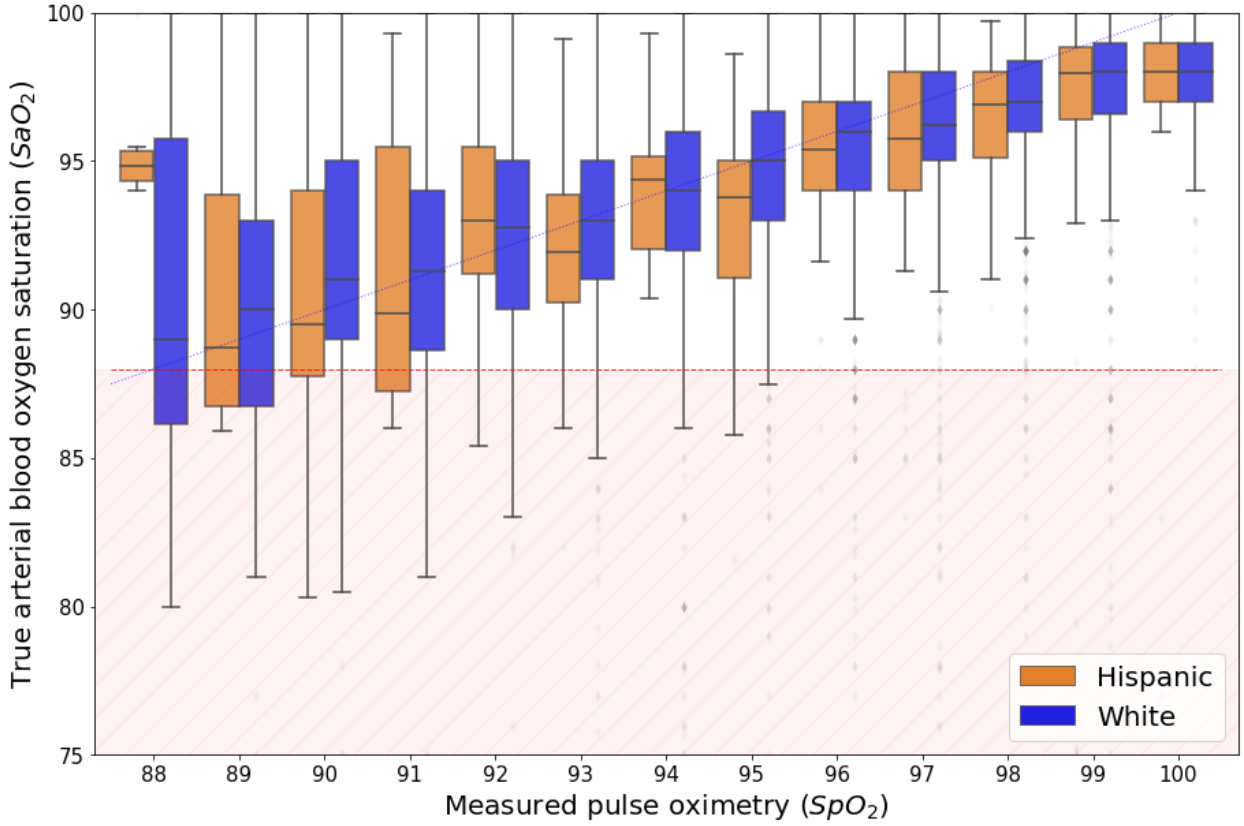

© 2021 Wong AI et al. *JAMA Network Open.*

cvSOFA = 1: Hispanic (n=959) compared with White (n=18,112)

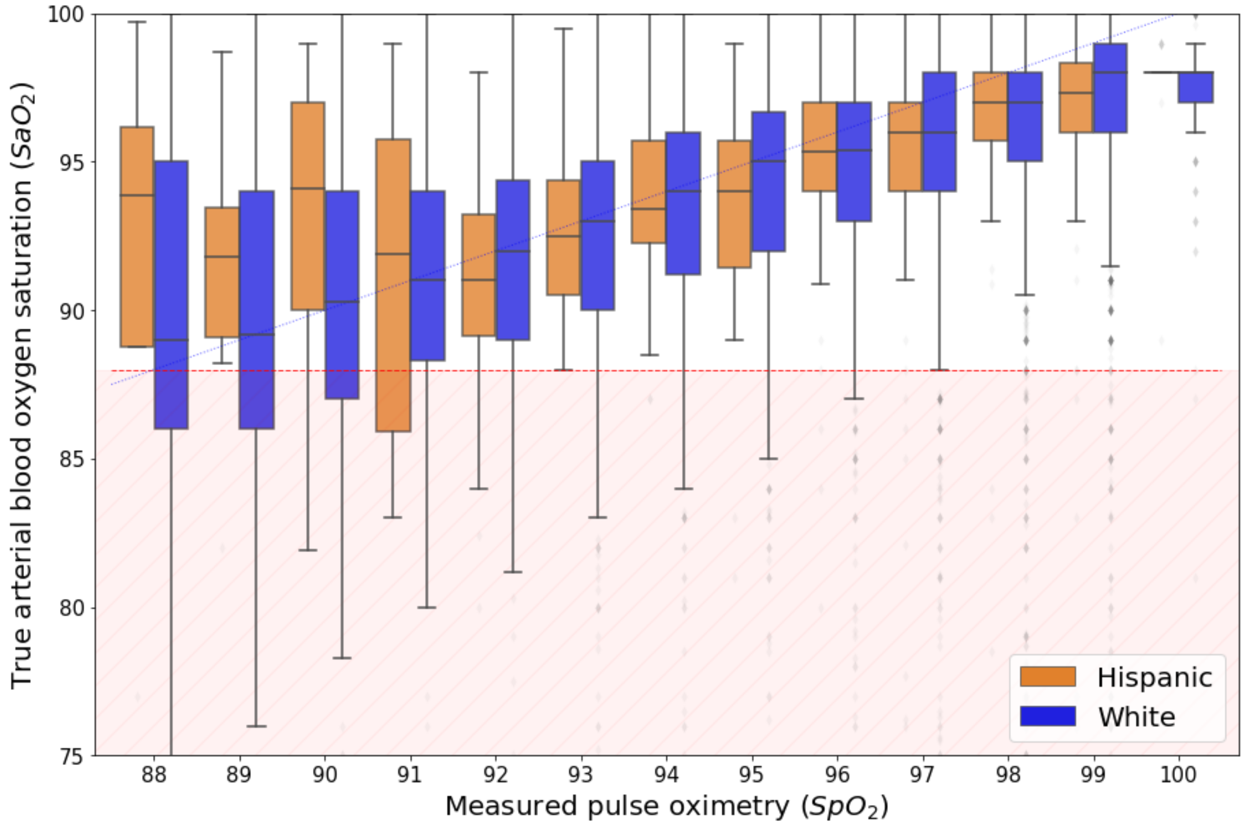

© 2021 Wong AI et al. *JAMA Network Open.*

cvSOFA = 2: Hispanic (n=16) compared with White (n=681)

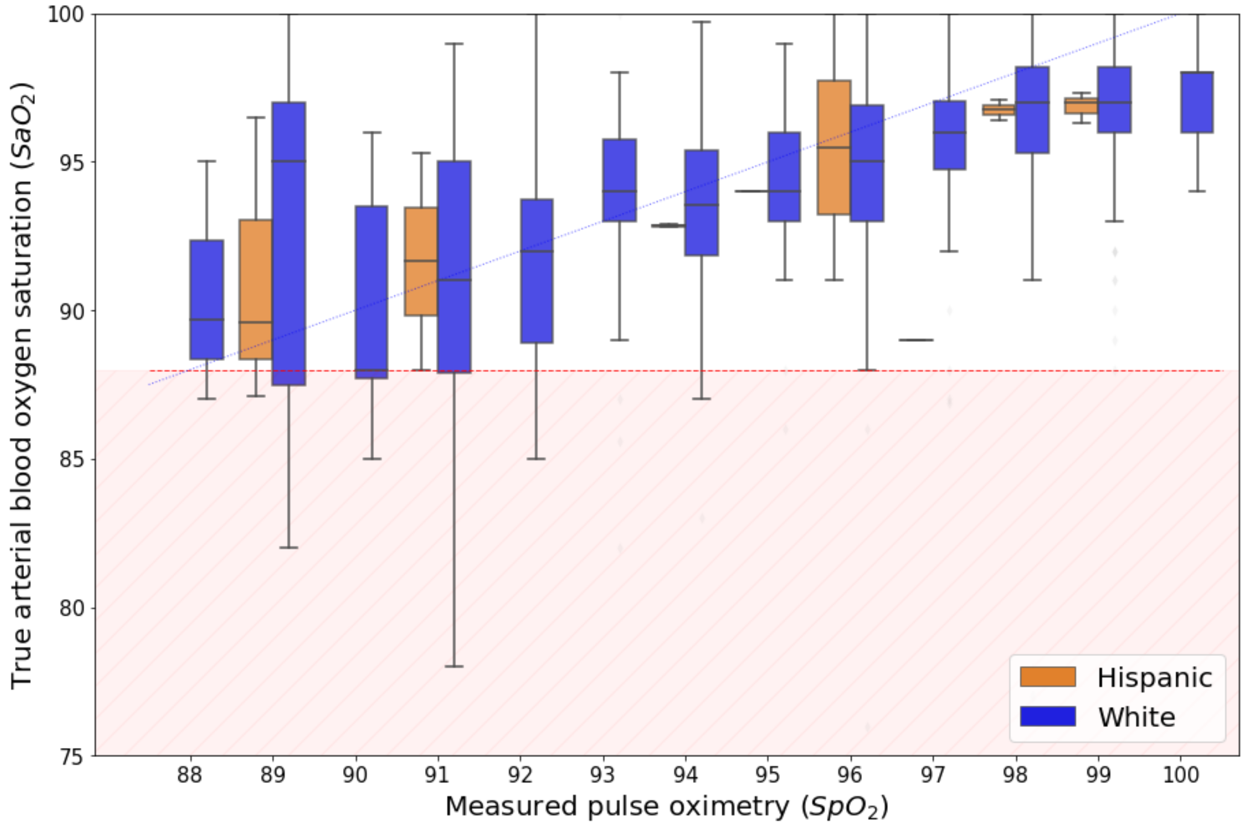

© 2021 Wong AI et al. *JAMA Network Open.*

cvSOFA = 3: Hispanic (n=119) compared with White (n=1,955)

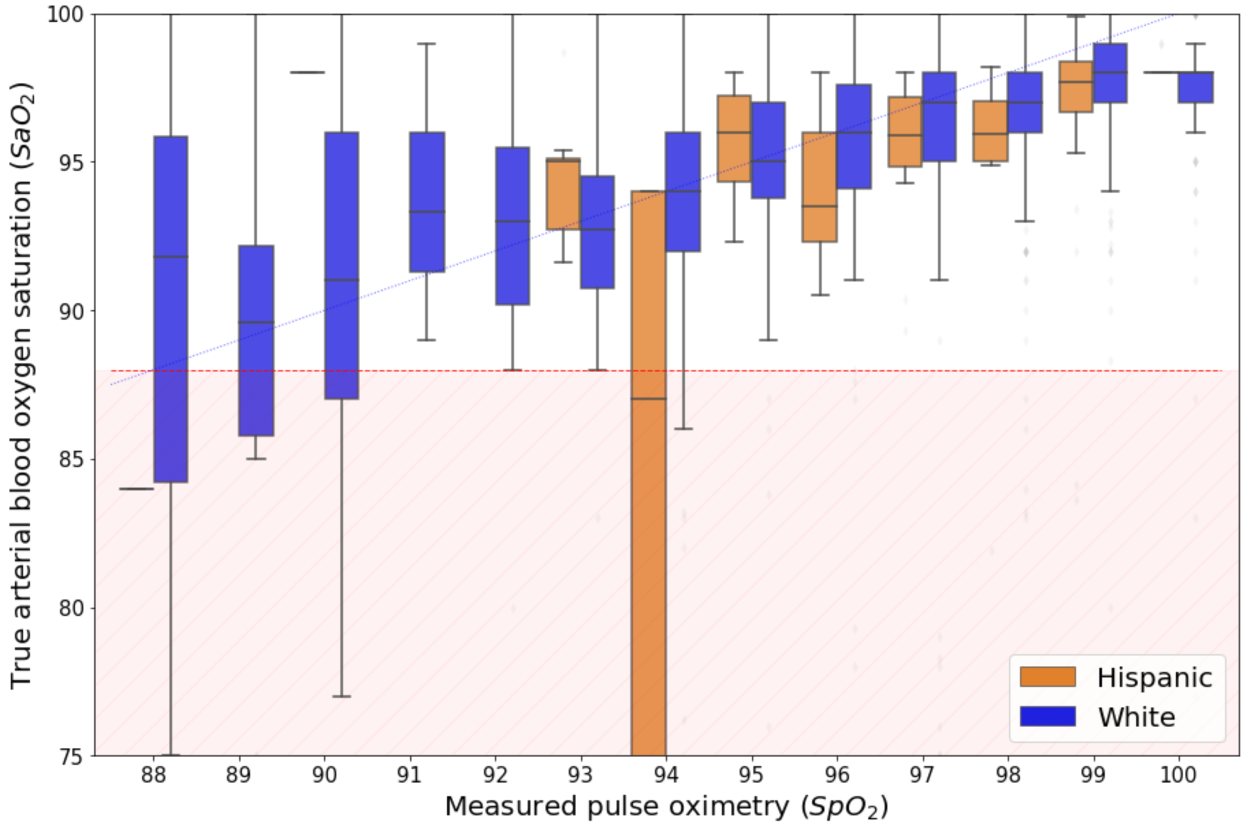

© 2021 Wong AI et al. *JAMA Network Open.*

cvSOFA = 4: Hispanic (n=153) compared with White (n=2,487)

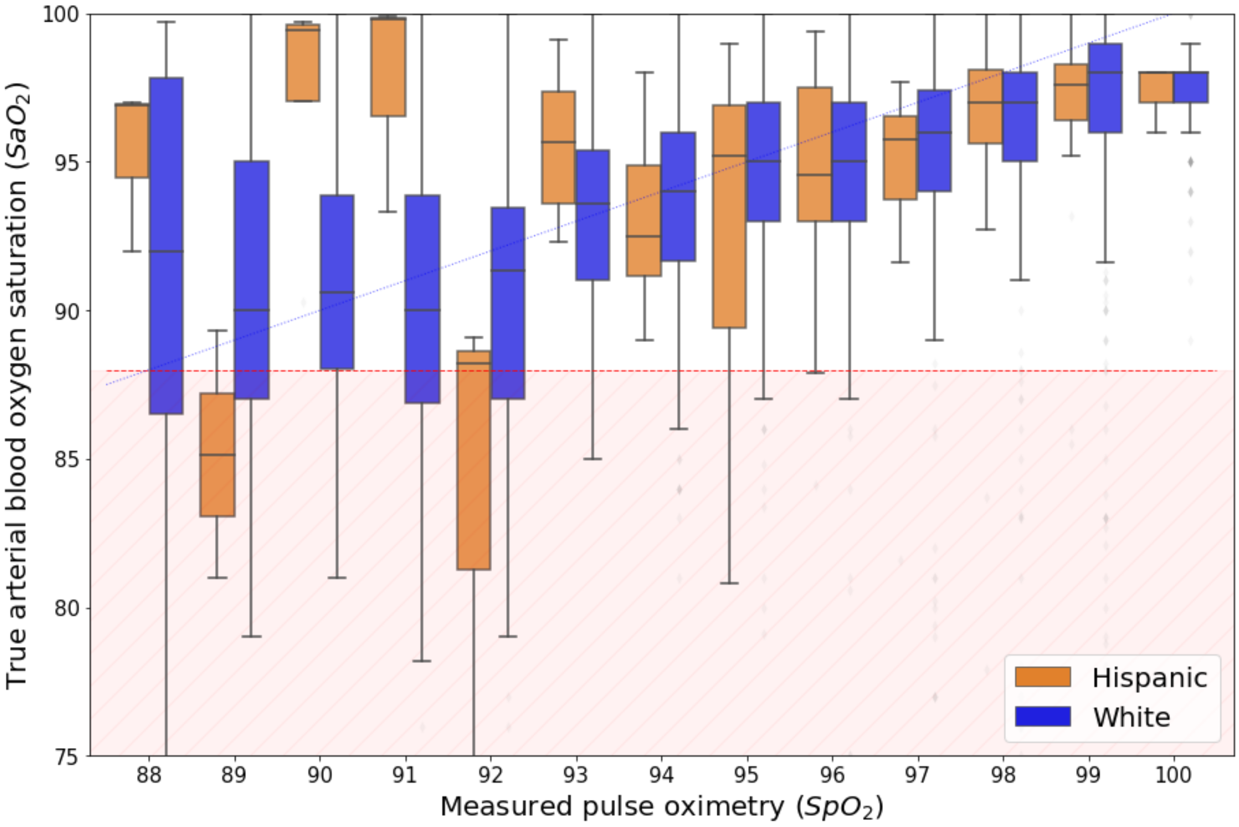

© 2021 Wong AI et al. *JAMA Network Open.*

eFigure 6. Directed Acyclic Graph for Race and Ethnicity, Hidden Hypoxemia, Organ Dysfunction, and Mortality

This directed acyclic graph (DAG) is a proposed graph suggesting causality and confounders for race-ethnicity, hypoxemia, organ dysfunction, and mortality.

Race-ethnicity, through skin color, appears to influence hidden hypoxemia.
Race-ethnicity may be directly associated with organ dysfunction, but this may also be influenced by the presence of hidden hypoxemia.
In addition to hypoxemia and organ dysfunction, race-ethnicity is a factor that could influence mortality.

More work must be done to elucidate the causal mechanisms.

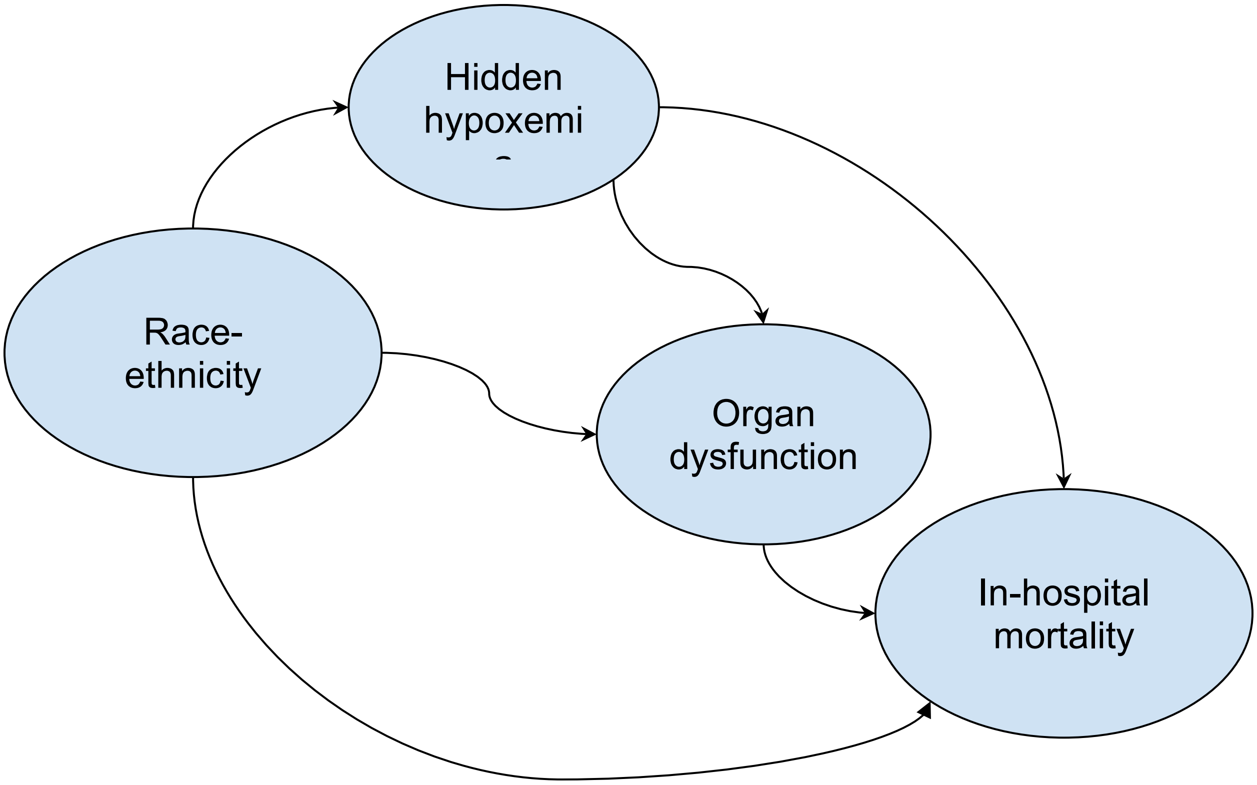

© 2021 Wong AI et al. *JAMA Network Open.*

eTable 1. Literature Review

The table presents an extensive review of the literature on pulse oximetry, skin pigmentation, and race-ethnicity.

**Author Year**	**Population/Settings /Sample**	**Method**	**Results** Descriptive: Bias: Precision (SD of bias):	**Notes/comments from papers**
[1] **ICU STUDIES IN CRITICALLY ILL ADULTS –SKIN COLOR NOT INCLUDED AS A VARIABLE**				
Seguin et al (2000) [1]	ICU – surgical, France<br>MV>24 hours<br>N=33 (two groups)<br>Group 1: 64 SaO_2-SpO_2 data pairs n=19<br>Group 2: 47 SaO_2-SpO_2 data pairs n=14<br><br>Exclusion Criteria:<br>- Bilirubin>40 mcg/mL<br>- Hemoglobin<9 g/dL<br>- Black skin<br>- No arterial line	A prospective observational study over six months.<br><br>Tests with two different pulse oximeter brands, described as Group 1 and 2.	Descriptive (Group 1):<br>- SaO_2 ranged from 87% to 98%.<br>- SpO_2 ranged from 92% to 100%.<br>Bias measurement: SaO_2 minus SpO_2*<br>Bias mean difference: -1.9%<br>Precision: 1.87%<br>Descriptive (Group 2):<br>- SaO_2 ranged from 87% to 99%.<br>- SpO_2 ranged from 92% to 100%.<br>Bias mean difference: -2.49%<br>Precision: 2.11%<br><br>Bland-Altman Plots: Yes.	An SpO_2 of 96% was necessary in this sample to ensure the $SaO_2 \geq 90\%$.<br><br>SpO_2 overestimated SaO_2.<br><br>Excluded patients with black skin.
Van de Louw et al. (2001)[2]	ICU – medical; France.<br>N=102<br>323 SpO_2-SaO_2 data pairs<br>Spont. Resp n=200<br>MV n=123<br><br>Exclusion Criteria:<br>-	A prospective observational study; convenience sample, consecutive patients.<br><br>Tests with three different pulse oximeter brands; multiple subgroup analyses.	Descriptive:<br>SaO_2 range from 91% to 100%.<br>SpO_2 range from 90% to 100%.<br>Bias measurement: SpO_2 minus SaO_2<br>Bias: -0.02%<br>Precision: 2.1%<br>Bland-Altman Plot: Yes	An SpO_2 of 94% was necessary in this sample to ensure the $SaO_2 \geq 90\%$.<br><br>Accuracy of SpO_2 appeared to be influenced by type of pulse oximeter, hypoxemia, and need for vasoactive medications.<br>No difference in bias between MV and spontaneous respirations in this sample.

© 2021 Wong AI et al. *JAMA Network Open.*

				Did not classify by skin color.
Perkins et al. (2003) [3]	ICU – mixed<br>N=41 (male=22)<br>1,085 SpO_2-SaO_2 data pairs over two months<br><br>Exclusion Criteria:<br>- Bilirubin>40 mcg/mL<br>- Smoke inhalation history	A prospective observational study; convenience sample, consecutive patients over 2 months<br><br>Tests with one SpO_2 sensor brand.	Descriptive:<br>SaO_2 mean ± SD: 95.9% (± 2.4%)<br>SpO_2 mean ± SD: 94.6% (± 2.7%)<br>Bias measurement: SaO_2 minus SpO_2<br>Bias: 1.34%<br>Precision: -<br>Bland-Altman Plots: Yes.<br><br>1085 paired readings (n=41) demonstrated only moderate correlation (r=0.606; p<0.01) between changes in SpO_2 and SaO_2.	The pulse oximeter overestimates changes in SaO_2.<br>Greater variations as saturations fall.<br><br>Less than 5% of data were SpO_2 below 90%.<br><br>Neither anemia nor acidosis altered the relation between SpO_2 and SaO_2 clinically.<br><br>Did not classify by skin color.
Wilson et al (2010) [4]	ED patients with septic shock admitted to three ICUs<br>N=88; UK<br><br>Exclusion Criteria:<br>- Hgb<7g/dL<br>- CO exposure<br>- Smoke inhalation	A retrospective cohort over 12 months.<br>Tested one SpO_2 brand.	Descriptive:<br>Mean SaO_2 90.2% (± 9.7%)<br>Mean SpO_2 93.9% (± 4.8%)<br><br>Bias measurement: SpO_2 minus SaO_2<br>Bias: 2.75%<br>Precision (SD): 3.1%<br>Bland-Altman Plot: Yes.<br><br>Sub-Group based on hypoxemia or not<br>Bias non-hypoxemia (SaO_2>90%) 1.89%<br>Bias with hypoxemia (SaO_2<90%) 4.92%<br>(difference statistically significant p<0.004).	Pulse oximetry overestimates ABG-determined SaO_2 by a mean of 2.75% in patients with severe sepsis and septic shock.<br><br>Accuracy of SpO_2 was not affected by acidosis, hyperlactatemia, anemia, or vasoactive drugs in this cohort.<br>Subgroup analysis indicated pulse oximeter readings were less accurate with hypoxemia (SaO_2<90).<br><br>Did not classify by skin color.
Singh et al. (2017) [5]	ICU – medical, India<br>N=129<br><br>Exclusion criteria:<br>- Venous Blood Gas<br>- Pigmented nails<br>- Methemoglobinemia<br>- Recent IV contrast	Prospective cohort study over two years.<br><br>Convenience ICU sample<br>Tested two SpO_2 brands<br><br>ABG and SpO_2 were obtained at the same time.	Descriptive:<br>Mean SaO_2 93.88% (± 8.68%)<br>Mean SpO_2 95.33% (± 8.33%)<br>Bias measurement equation: unclear<br>Pulse Oximeter #1<br>o Bias: 2.49%<br>o Precision (SD): 2.99%<br><br>Pulse Oximeter #2<br>o Bias: 0.46%	Pulse oximetry overestimated arterial oxygen saturation (SaO_2) by 1.44% (positive bias).<br>Bias increased with lower SaO_2.<br><br>Bias increased with higher lactate levels (p=0.16) and hypoxemia (lower SaO_2).<br><br>Did not classify by skin color.

© 2021 Wong AI et al. *JAMA Network Open.*

			○ Precision (SD): 1.68<br><br>Bland-Altman Plot: Yes	
Nisar et al. (2020)[6]	ICU on V-V ECMO<br>USA<br>N=40	Retrospective observational Study over 5 years<br>V-V-ECMO	"Of the 1,496 ABG analyses, elevated COHb (>3%) occurred in 602 (40.24%) samples."	"On average, SpO_2 over-estimated SaO_2 during the entire timeframe of VV-ECMO support."<br><br>Did not classify by skin color.
**ICU STUDIES IN CRITICALLY ILL ADULTS –SKIN COLOR IS INCLUDED AS A VARIABLE**				
Bothma et al. (1996) [7]	ICU – mixed; South Africa. N=100 stable critically ill patients with darkly pigmented skin; arterial line for clinical care.<br><br>Skin pigmentation was objectively quantified.<br><br>Exclusion Criteria:<br>- Hgb<7g/dL<br>- Carboxyhemoglobin <2%<br>- Methemoglobin <2%<br><br>Number of SaO_2-SpO_2 pairs that were analyzed was not listed.	A prospective observational study, convenience sample. Patients were recruited based on the appearance of darkly pigmented skin. Skin pigmentation was verified with a ***portable reflectance spectrophotometer***.<br><br>ABG by co-oximetry<br><br>Pulse Oximeter (SpO_2) tested 3 brands on finger, and one of the brands on the ear	Descriptive:<br>- SaO_2 range 87.8% - 99.2% (median 96%);<br>- SpO_2 range 86% to 100%.<br>Bias measurement equation: unclear.<br><br>Bias: -1.0 to 1.2 (varied by brand)<br>Precision: 1.9 to 2.4 (varied by brand)<br>Bland- Altman Plot: Yes	Per authors, Pulse Oximeter manufacturers "claim ± 2% (SD) accuracy over a range of 70% - 100% saturation."<br>Results were within this range.<br><br>Hypoxemia Note<br>Per authors, the pulse oximeter readings at SaO_2<92% tended to be lower than co-oximeter ABG values. A few measurements fell below 2 SD. Insufficient number of values at low SaO_2 range to make a conclusive statement as only five SaO_2 readings were <92% - the total number of analyses was not provided.<br><br>Study participants were selected for darkly pigmented skin.<br><br>Skin pigment color addressed.
Adler et al. (1998) [8]	ED – community hospital; USA.<br>N=284	A prospective observational study, convenience sample, over four months.	Descriptive:<br>SaO_2 range 50% to 99%; mean SaO_2 91% (± 6%).<br>SpO_2 range 66% to 100%; mean SpO_2 94% (± 5%).	Overall, pulse oximetry (SpO_2) overestimated SaO_2 (ABG) but no significant difference was reported for skin color.

© 2021 Wong AI et al. *JAMA Network Open.*

		Participants' skin pigment was classified into three groups using the ***Munsell Color tile system***.<br><br>ABGs measured by co-oximetry.<br><br>Pulse Oximeter (SpO_2) tested 1 brand.	Bias:<br>o Light skin: 2.5%;<br>o Intermediate skin: 2.8%;<br>o Dark skin: 2.2%.<br><br>Precision (SD):<br>o Light skin: 4.6%;<br>o Intermediate skin: 5.2%;<br>o Dark skin: 3.7%.<br><br>Bland-Altman plot: No.<br><br>"Bias values were not significantly different across the 3 groups (p=0.79). Although precision was of borderline significance (p=0.051) no dose-response relationship was seen."<br><br>Pulse oximetry signal failure was <1%.	It was not reported how many participants were in each of the three groups, although percentages were reported as:<br>- light (51%);<br>- intermediate (37%);<br>- dark (12%).<br><br>Skin pigment color addressed.
Ebmeier et al. (2018) [9]	ICU – mixed medical and surgical;<br>Australia & New Zealand.<br>N=394 (two ICUs)<br>o n=210 (60%) M. Vent<br>o n=116 Suppl. O_2<br>o n=25 (7%) Room air	Prospective cohort observational study over approximately 12 months in two different ICUs.<br><br>The ***Fitzpatrick scale*** was used to categorize skin color:<br>o Light (1 or 2);<br>o Medium (3 or 4);<br>o Dark (5 or 6).<br><br>Two pulse oximeter brands tested from either ear, finger or toe.	Descriptive:<br>Mean (SD) SaO_2 95.7% (± 2.7%)<br>Mean (SD) SpO_2 95.6% (± 3.0%)<br>Bias measurement: SaO_2 minus SpO_2<br><br>Significant differences between light and dark skin, and light and intermediate skin.<br>Also differences between the pulse oximeter brands.<br><br>Bland-Altman Plot: Yes.	Found no statistically significant difference in paired SaO_2 and SpO_2 measurements.<br><br>Did find a difference related to skin color and to pulse oximeter brand.<br><br>Skin pigment color addressed.
Sjoding et al (2021) [10]	Adult patients receiving supplemental O_2 at University of Michigan Hospital.<br>o White: n=1,333<br>o Black: n=276<br><br><br>Adult patients in multicenter cohort database	University of Michigan Hospital - 6 months in 2020<br><br>Multicenter cohort 2014-2015<br><br>Total: Analyzed 10,789 pairs of SpO_2 and SaO_2	Bias or Precision were not described.<br><br>In the University of Michigan Hospital cohort, for patients with SpO_2 92% to 96% the paired SaO_2 was:<br><88% in 88 of 749 Black patients (11.7%)<br><88% in 99 of 2,778 White patients (3.6%)	"In two large cohorts, Black patients had nearly three times the frequency of occult hypoxemia that was not detected by pulse oximetry as White patients."<br><br>Skin pigment color addressed.

© 2021 Wong AI et al. *JAMA Network Open.*

	○ White: n=7,342<br>○ Black: n=1,050		In the Multicenter cohort, for patients with SpO_2 92% to 96% the paired ABG-SaO_2 was:<br><88% in 160 of 939 Black patients (17%)<br><88% in 546 of 8,795 White patients (6%)	
**HEALTHY ADULT VOLUNTEERS – EXPERIMENTAL STUDIES -–SKIN COLOR <u>IS</u> INCLUDED AS A VARIABLE**				
Bickler et al. (2005) [11]	Healthy adult volunteers; USA.<br>N=21<br>Subgroups:<br>n=11 skin pigment dark<br>n=10 skin pigment light	Experimental Comparative Study<br>-Three pulse oximeter brands attached to each participant.<br>- Radial arterial line *in situ*.<br>- Participants semi-supine with HOB at 30 degrees.<br>-Participants were breathing a mixture of air-nitrogen-carbon dioxide via a mouthpiece and partial re-breathing circuit – obtained end-tidal gases also.<br>- "A computer used end-tidal oxygen and carbon dioxide concentrations determined by mass spectrometry to estimate breath-by-breath SaO_2, from which an operator adjusted inspired gas to rapidly achieve 2- to 3-min stable plateaus of desaturation"	<u>Descriptive:</u><br>Data on bias was presented for a range of SaO_2 values (60-70%; 70-80%; 80-90% and 90-100%) compared by skin pigment light or dark (See Table 1 in paper) for 1,067 data points.<br><br><u>Bias measurement:</u> SpO_2 minus SaO_2<br><br><u>All</u> values across a SaO_2 range of 50-100%<br>o <u>Bias:</u> Light skin 0.21 (SD ± 2.0%).<br>o <u>Bias:</u> Dark skin 1.13 (SD ± 2.3%).<br><br>P<0.0001 between light and dark<br>o <u>Precision:</u> Light skin 2.0%<br>o <u>Precision:</u> Dark skin 2.13%<br><br>Bland Altman Plot: No<br><br>Graphs plotted bias versus SaO_2	"The effect of skin pigment on bias increased as SaO_2 decreased."<br><br>Skin pigment color addressed.<br><br>"The three tested pulse oximeters overestimated arterial oxygen saturation during hypoxemia in dark-skinned individuals."
Feiner et al (2007) [12]	Healthy Adult Volunteers<br>N=36<br>(19 males, 17 females)<br>Range of skin tones	Experimental Comparative Study<br>Semi-supine position. Participants breathed air-nitrogen-CO_2 mixtures<br>via a mouthpiece to rapidly achieve 2- to 3-min stable plateaus of SaO_2.<br>Compared SpO_2 (pulse oximetry) with SaO_2 (by Radiometer OSM-3) at different SaO_2 range levels.	<u>Bias measurement:</u> SpO_2 minus SaO_2<br><br>Bias at different saturation levels.<br>At 70%-80% range –<br><u>Bias:</u> Clip1 1.58%; Clip2 2.59%;<br><u>Bias:</u> Disp1 -0.6%; Disp2 2.43%<br><br>Bias affected by skin pigment and by the type of sensor.<br>Skin classification:	Darker skin increased bias at low SaO_2; greater bias was seen with adhesive/disposable sensors than with the clip-on types.<br><br>Up to 10% differences in saturation estimates were found among different instruments in dark-skinned participants at low SaO_2.

© 2021 Wong AI et al. *JAMA Network Open.*

		- 4 types of sensors, 2 were clip on, and 1 was disposable.<br>- multivariate model	○ Light;<br>○ Intermediate;<br>○ Dark.<br><br>Bland Altman Plot: No.<br><br>Graphs plotted mean bias versus SaO_2 at different SaO_2 ranges by type of sensor	Follow-up study from same group as Bickler et al (2005).<br><br>Skin pigment color addressed.

Definitions

Descriptive: SaO_2 and SpO_2 percentages: range, mean, standard deviation (SD) as reported by each study.
Bias: The mean differences between two values. Bias is a measure of trueness. SaO_2 is the gold-standard.
Some studies used SaO_2 minus SpO_2. However, other studies used SpO_2 minus SaO_2 to report bias.
Some studies did not state how bias was calculated and it was not clear from the paper whether SaO_2 - SpO_2 or the reverse (i.e., SpO_2 - SaO_2) was used; in the table, we qualify this uncertainty as follows: "bias measurement method unclear".
Precision: The standard deviation (SD) of the bias.

© 2021 Wong AI et al. *JAMA Network Open.*

eTable 2. SOFA Score Components, as per Vincent et al, 1996

For the convenience of the reader, the components of the SOFA score as per Vincent et al 1996.

SOFA score	1	2	3	4
**Respiration (rSOFA)**				
PaO_2/FiO_2 (mm Hg)	<400	<300	<220	<100
**Coagulation**				
Platelets ×10^3/mm^3	<150	<100	<50	<20
**Liver**				
Bilirubin (mg/dL)	1.2-1.9	2.0-5.9	6.0-11.9	>12.0
**Cardiovascular (cvSOFA)**				
Hypotension	MAP <70	Dopamine ≤5 or dobutamine (any)	Dopamine >5 or norepinephrine ≤0.1	Dopamine >15 or norepinephrine >0.1
**CNS**				
Glasgow Coma Score	13-14	10-12	6-9	<6
**Renal**				
Creatinine (mg/dL) or urine output (mL/d)	1.2-1.9	2.0-3.4	3.5-4.9 or <500	>5.0 or <200

© 2021 Wong AI et al. *JAMA Network Open.*

eTable 3. Other Patient Characteristics

eTable 3a. Patient characteristics for All ABGs examined

Patient characteristics for all ABGs examined, which represents characteristics of patients with a SpO_2 within the five minutes preceding the ABG, based on all ABGs in their hospital encounters. When applicable, standard errors (SE) are provided. They were obtained using simple bootstrap with 100 iterations.

	All ABGs in hospital encounter SpO2 within 30 minutes of ABG	
Total patients (n)	121,099	
Total hospital encounters (n)	141,600	
Total ABGs (n)	679,909	
Total SpO2-SaO2 pairs (n)	5,435,144	
Sex (n and % female)	61,628	43.5%
Age (mean)	61.26 ± 16.37	
SaO2 (mean ± standard error)	94.95 ± 6.20	
SpO2 (mean ± standard error)	96.61 ± 2.81	
Cardiovascular SOFA (mean ± standard error)	0.72 ± 0.0009	
Race-Ethnicity (n, %)		
Asian	3,249	2.3%
Black	49,053	34.6%
Hispanic	3,426	2.4%
White	85,872	60.6%
Hospital encounters (n)		
eICU-CRD	42,238	29.8%

© 2021 Wong AI et al. *JAMA Network Open.*

MIMIC-III	5,150	3.6%
MIMIC-IV	12,256	8.7%
Emory	59,044	41.7%
Grady	22,912	16.2%
**ABGs (n, %)**		
eICU-CRD	171,322	25.2%
MIMIC-III	26,822	3.9%
MIMIC-IV	63,084	9.3%
Emory	283,513	41.7%
Grady	135,168	19.9%
**SaO2-SpO2 pairs (n, %)**		
eICU-CRD	1,852,528	34.1%
MIMIC-III	44,371	0.8%
MIMIC-IV	96,885	1.8%
Emory	3,227,305	59.4%
Grady	214,055	3.9%

© 2021 Wong AI et al. *JAMA Network Open.*

eTable 3b. Patient characteristics for first ABGs examined among patients of other/missing race/ethnicity

Patient characteristics for all patients with missing or race/ethnicity not conforming to Asian, Black, Hispanic, and White (n = 6,269) by with and without occult hypoxemia. Note that in-hospital mortality and short term organ dysfunction outcomes continue to be elevated in patients with occult hypoxemia.

	others				
	Occult hypoxemia		No hypoxemia		p
n	251	4.0%	6018	96.0 %	
Age (mean)	59.21 ± 1.4		60.57 ± 0.27		<0.001
Sex (% female)	111	44.2%	2445	40.6 %	0.26
% male	140	55.8%	3573	59.4 %	
	Long-term clinical outcomes				
Hospital LoS (mean, in days) (survivors only)	11.28 ± 1.18		12.43 ± 0.26		<0.001
in-hospital death	71	28.3%	1224	20.3 %	0.00
	At the time of ABG				
cvSOFA (mean ± standard error)	1.19 ± 0.13		1.16 ± 0.03		<0.001
rSOFA (mean ± standard error)					
SOFA (mean ± standard error)	5.57 ± 0.4		5.72 ± 0.08		<0.001

© 2021 Wong AI et al. *JAMA Network Open.*

	Short-term organ dysfunction outcomes (24 hours after the ABG was drawn)		
cvSOFA (mean ± standard error)	1.63 ± 0.14	1.44 ± 0.03	<0.001
rSOFA (mean ± standard error)	1.85 ± 0.15	1.42 ± 0.03	<0.001
SOFA (mean ± standard error)	7.39 ± 0.42	6.41 ± 0.09	<0.001

© 2021 Wong AI et al. *JAMA Network Open.*

eTable 3c. Demographic characteristics for patients with and without ABGs

	Asian			Black			Hispanic			White		
	Received ABG(s)	No ABGs	p	Received ABG(s)	No ABGs	p	Received ABG(s)	No ABGs	p	Received ABG(s)	No ABGs	p
n	2188 (7.7%)	26367 (92.3%)		38449 (12.6%)	265603 (87.4%)		3848 (9.6%)	36145 (90.4%)		63323 (14%)	389998 (86%)	
In-hospital mortality	212 (9.7%)	665 (2.5%)		2764 (7.2%)	3539 (1.3%)		522 (13.6%)	796 (2.2%)		8629 (13.6%)	22114 (5.7%)	
Age	64.38 ± 0.58	36.43 ± 0.27	< 0.001	58.14 ± 0.1	51.68 ± 0.05	< 0.001	58.3 ± 0.41	43.87 ± 0.15	< 0.001	65.65 ± 0.11	58.41 ± 0.06	< 0.001
Length of Stay (days)	11.93 ± 0.56	5.07 ± 0.06	< 0.001	13.14 ± 0.13	5.57 ± 0.02	< 0.001	12.89 ± 0.43	5.22 ± 0.06	< 0.001	10.26 ± 0.05	5.69 ± 0.01	< 0.001
Creatinine	1.76 ± 0.06	1.53 ± 0.03	< 0.001	2.39 ± 0.02	2.04 ± 0.01	< 0.001	1.93 ± 0.05	2.1 ± 0.03	< 0.001	1.51 ± 0.01	1.34 ± 0.0	< 0.001
Lactate	2.88 ± 0.1	2.15 ± 0.04	< 0.001	2.93 ± 0.02	2.13 ± 0.01	< 0.001	2.75 ± 0.08	2.19 ± 0.03	< 0.001	2.63 ± 0.02	2.16 ± 0.01	< 0.001
SOFA	6.58 ± 0.2	4.53 ± 0.07	< 0.001	7.07 ± 0.07	4.81 ± 0.03	< 0.001	7.09 ± 0.1	4.84 ± 0.05	< 0.001	6.97 ± 0.02	4.66 ± 0.01	< 0.001
CVSOFA	1.26 ± 0.04	0.9 ± 0.02	< 0.001	1.19 ± 0.02	0.75 ± 0.01	< 0.001	1.35 ± 0.03	0.89 ± 0.01	< 0.001	1.3 ± 0.01	0.89 ± 0.0	< 0.001
RSOFA	1.29 ± 0.07	0.27 ± 0.02	< 0.001	1.36 ± 0.03	0.35 ± 0.01	< 0.001	1.4 ± 0.04	0.35 ± 0.01	< 0.001	1.29 ± 0.01	0.32 ± 0.0	< 0.001

© 2021 Wong AI et al. *JAMA Network Open.*

eTable 4. Characterization of Variable Missingness Across the 5 EHR Data Sets

Missingness overall and per dataset. A value of 0.0% indicates that no data was missing. Note that SOFA scores in eICU-CRD, MIMIC-III, and MIMIC-IV datasets were calculated by carrying forward the value of its six components. Thus, there are situations where there may not be a cvSOFA value mapping to the hour preceding the ABG, in which case a SOFA score is calculated with the carried-forward cvSOFA value (instead of a newly updated cvSOFA value).

	all	*eICU-CRD*	*MIMIC-III*	*MIMIC-IV*	*Emory*	*Grady*
**Age**	0.0%	0.0%	0.0%	0.0%	0.0%	0.0%
**Sex**	0.0%	0.0%	0.0%	0.0%	0.0%	0.0%
**rSOFA**	52.7%	9.0%	0.0%	0.0%	100.0%	100.0%
**cvSOFA**	52.9%	9.0%	4.3%	1.6%	100.0%	100.0%
**SOFA**	52.7%	1.1%	0.0%	0.0%	100.0%	100.0%
**In-hospital mortality**	0.4%	1.0%	0.0%	0.0%	0.0%	0.0%
**Length of stay**	0.1%	0.0%	0.0%	0.0%	0.9%	0.7%
**Creatinine before ABG**	19.3%					
**Creatinine after ABG**	47.3%					
**Lactate before ABG**	45.2%					
**Lactate after ABG**	77.1%					

© 2021 Wong AI et al. *JAMA Network Open.*

eTable 5. SpO_2 Variability for All SpO_2 Values Within the 5 Minutes Preceding the ABG Measurement

The table presents the variability in SpO2 measurements contained within a five-minute interval preceding the ABG, as measured by the difference between the two most extreme pulse oximetry values. Results are aggregated across the five databases and stratified by race-ethnicity.

Racial-ethnic subgroup	SpO_2 variability (mean difference between minimum and maximum value ± standard error)
**All**	0.627 ± 1.87%
**Asian**	0.488 ± 1.62%
**Black**	0.505 ± 2.08%
**Hispanic**	0.627 ± 1.87%
**White**	0.672 ± 1.71%

eTable 6. Percentage of Encounters With Arterial Blood Gases, by Race and Ethnicity

This table describes the percentage of encounters in all datasets (eICU-CRD, MIMIC-III, MIMIC-IV, Emory, and Grady) that have at least 1 ABG by race-ethnicity compared to all encounters examined. Chi square test of this distribution is $p < 0.001$.

Because of the composition of the Emory and Grady datasets, this table includes both ICU and non-ICU patients.

Race-ethnicity	ABG	No ABG	Total (n)	Observed (%)	Expected (%)
**Asian**	3249	92564	95813	3.4%	3.9%
**Black**	49053	1732815	1781868	2.8%	3.9%
**Hispanic**	3426	176191	179617	1.9%	3.9%
**White**	85872	1446620	1532492	5.6%	3.9%
**Total**	141600	3448190	3589790		

This table describes the percentage of encounters in eICU-CRD, MIMIC-III, and MIMIC-IV that have at least 1 ABG by race-ethnicity compared to all encounters examined. As only these datasets have SOFA scores calculated, Chi square test of this distribution is $p < 0.001$.

Because of the composition of these datasets, this table includes only ICU patients.

						at the time of the first ABG		
Race-ethnicity	ABG	No ABG	Total (n)	Observed (%)	Expected (%)	rSOFA (mean ± standard error)	cvSOFA (mean ± standard error)	SOFA (mean ± standard error)
**Asian**	790	28669	29459	2.7%	6.4%	0.97 ± 0.06	0.99 ± 0.05	5.05 ± 0.17
**Black**	4260	102054	106314	4.0%	6.4%	0.90 ± 0.03	0.89 ± 0.02	5.31 ± 0.08
**Hispanic**	2009	36951	38960	5.2%	6.4%	0.97 ± 0.04	1.05 ± 0.04	5.37 ± 0.1
**White**	38004	486374	524378	7.2%	6.4%	0.89 ± 0.01	1.01 ± 0.01	5.29 ± 0.03
**Total**	45063	654048	699111					

© 2021 Wong AI et al. *JAMA Network Open.*

eTable 7. Rate of ABG Measurements Obtained Throughout a Hospital Encounter by Race and Ethnicity, Stratified by Cardiovascular SOFA Score

cvSOFA	ethnicity	N	Mean	SD	SE	95% Conf.	Interval
0	Asian	623	0.88	1.21	0.05	0.78	0.97
0	Black	3983	0.72	0.98	0.02	0.69	0.75
0	Hispanic	1481	0.83	1.56	0.04	0.75	0.91
0	White	27146	0.81	1.16	0.01	0.79	0.82
1	Asian	1744	1.08	1.33	0.03	1.02	1.15
1	Black	7844	1.05	1.43	0.02	1.02	1.09
1	Hispanic	4596	0.96	1.04	0.02	0.93	0.99
1	White	72836	1.02	1.29	0.00	1.01	1.03
2	Asian	62	2.73	2.52	0.32	2.09	3.37
2	Black	402	1.27	1.29	0.06	1.14	1.40
2	Hispanic	131	0.87	0.76	0.07	0.74	1.00
2	White	4183	1.36	1.60	0.02	1.31	1.41
3	Asian	183	1.08	1.33	0.10	0.89	1.28
3	Black	646	0.80	0.72	0.03	0.75	0.86
3	Hispanic	559	1.53	3.62	0.15	1.23	1.83
3	White	9116	1.08	1.19	0.01	1.06	1.10
4	Asian	278	1.43	1.53	0.09	1.25	1.61
4	Black	1318	1.19	1.26	0.03	1.12	1.26
4	Hispanic	910	2.20	4.19	0.14	1.93	2.48
4	White	14280	1.41	1.60	0.01	1.39	1.44

© 2021 Wong AI et al. *JAMA Network Open.*

eTable 8. Descriptive Statistics for All SaO_2-SpO_2 Pairs With SpO_2 of at Least 88%

Supplemental Table 8a. Descriptive statistics (mean, median, interquartile range), stratified by race-ethnicity

At each SpO_2 level, the mean, median, and IQR of SaO_2 are presented. These statistics are calculated by 100-iteration bootstrapping. Results are stratified by race-ethnicity.

	Mean SaO_2					***Median SaO_2***					***IQR for SaO_2***			
SpO_2	***Asian***	***Black***	***Hispanic***	***White***		***Asian***	***Black***	***Hispanic***	***White***		***Asian***	***Black***	***Hispanic***	***White***
**88**	90.58	88.76	93.11	89.91		88.90	90.10	94.80	90.00		9.15	10.13	3.85	9.10
**89**	90.81	89.10	89.14	89.53		89.50	90.10	89.40	90.00		7.95	9.20	6.25	7.00
**90**	90.27	90.03	92.64	90.53		89.60	91.60	92.00	91.00		5.85	7.40	9.10	7.00
**91**	91.14	90.72	90.12	91.14		91.00	91.50	91.45	92.00		3.05	6.60	9.05	6.00
**92**	91.54	90.77	91.47	91.93		92.50	92.00	91.80	92.50		3.60	6.10	5.70	5.00
**93**	92.43	91.51	92.14	92.49		92.55	93.00	93.00	93.20		4.45	5.60	4.00	4.40
**94**	92.80	92.40	93.44	93.33		93.60	93.90	94.00	94.00		4.57	5.10	3.00	4.00
**95**	93.40	92.98	93.09	93.94		95.00	94.60	94.00	95.00		4.00	4.90	4.80	3.80
**96**	95.14	93.53	94.08	94.70		95.85	95.10	95.10	96.00		3.20	4.00	3.10	3.00
**97**	94.48	94.48	94.91	95.32		96.10	96.00	96.00	96.30		3.30	3.40	3.50	3.00
**98**	95.71	95.40	95.89	96.07		97.00	96.90	97.00	97.00		2.60	3.00	2.70	2.40
**99**	96.38	95.92	96.51	96.82		97.80	97.30	97.50	98.00		2.70	2.60	2.40	2.60
**100**	97.97	97.21	96.72	98.01		98.60	98.30	98.00	98.70		1.40	2.10	1.00	1.20

© 2021 Wong AI et al. *JAMA Network Open.*

eTable 8b. Total number of patients per SpO_2 level and characterization of hidden hypoxemia incidence, stratified by race-ethnicity

	Count					*Count, SaO2<88%*					*%, SaO_2<88%*			
**SpO_2**	**Asian**	**Black**	**Hispanic**	**White**		**Asian**	**Black**	**Hispanic**	**White**		**Asian**	**Black**	**Hispanic**	**White**
**88**	11	216	15	521		4	77	2	169		36.36%	35.65%	13.33%	32.44%
**89**	18	258	27	612		5	87	10	187		27.78%	33.72%	37.04%	30.56%
**90**	31	383	31	932		10	98	5	203		32.26%	25.59%	16.13%	21.78%
**91**	35	462	48	1327		3	93	14	230		8.57%	20.13%	29.17%	17.33%
**92**	55	674	61	1912		9	119	10	224		16.36%	17.66%	16.39%	11.72%
**93**	92	840	87	2511		9	126	6	230		9.78%	15.00%	6.90%	9.16%
**94**	74	1162	121	3391		7	139	6	203		9.46%	11.96%	4.96%	5.99%
**95**	112	1593	145	4426		8	145	11	219		7.14%	9.10%	7.59%	4.95%
**96**	134	1859	241	5565		3	145	11	226		2.24%	7.80%	4.56%	4.06%
**97**	159	2217	319	6732		8	131	21	255		5.03%	5.91%	6.58%	3.79%
**98**	219	2959	406	8601		8	152	14	280		3.65%	5.14%	3.45%	3.26%
**99**	346	3555	610	11589		14	154	22	300		4.05%	4.33%	3.61%	2.59%
**100**	633	9854	286	9504		6	323	13	105		0.95%	3.28%	4.55%	1.10%

© 2021 Wong AI et al. *JAMA Network Open.*

eTable 9. Total Number of Patients per SpO_2 Level and Characterization of Hidden Hypoxemia Incidence, Stratified by Race and Ethnicity

The risk of occult hypoxemia is characterized by race-ethnicity. Each cell in the table represents the percentage of patients with occult hypoxemia ($SpO_2 \geq 88\%$, $SaO_2 < 88\%$) in the considered race/ethnicity subgroup for a given pulse oximetry level. The corresponding bootstrapped standard error of the mean (SE) for 100 iterations is also provided. The number (n) indicates the total number of patients in the considered race/ethnicity subgroup at that given pulse oximetry level (including patients with and without occult hypoxemia).

To concretely describe differential levels of occult hypoxemia risk by race-ethnicity, consider the risk at SpO_2=92%, which is often an oxygenation goal. In White patients, the risk of occult hypoxemia is 15.2%; this risk increases to 18.4% for Hispanic and Asian patients (a 20% higher relative risk), and up to 20.2% for Black patients (a 33% higher relative risk).

SpO_2	Asian (n=1,919)	Black (n=26,032)	Hispanic (n=2,397)	White (n=57,623)
88%	42.3% ± 19.0% (n=11)	37.8% ± 4.0% (n=213)	12.6% ± 12.0% (n=13)	36.5% ± 3.0% (n=495)
89%	29.5% ± 15.0% (n=17)	35.1% ± 4.0% (n=252)	45.6% ± 11.0% (n=24)	35.2% ± 3.0% (n=580)
90%	34.3% ± 11.0% (n=30)	27.9% ± 3.0% (n=368)	26.1% ± 10.0% (n=27)	25.7% ± 2.0% (n=884)
91%	11.6% ± 6.0% (n=35)	22.1% ± 3.0% (n=456)	38.5% ± 10.0% (n=46)	21.0% ± 2.0% (n=1298)
92%	18.5% ± 6.0% (n=53)	20.2% ± 2.0% (n=661)	18.4% ± 6.0% (n=57)	15.2% ± 1.0% (n=1834)
93%	11.0% ± 5.0% (n=90)	16.3% ± 2.0% (n=825)	8.7% ± 4.0% (n=83)	10.6% ± 1.0% (n=2451)
94%	7.6% ± 4.0% (n=72)	12.1% ± 1.0% (n=1145)	5.6% ± 3.0% (n=114)	7.2% ± 1.0% (n=3291)
95%	7.3% ± 3.0% (n=110)	10.1% ± 1.0% (n=1573)	7.9% ± 3.0% (n=141)	5.7% ± 0.0% (n=4329)
96%	2.4% ± 2.0% (n=132)	8.2% ± 1.0% (n=1824)	5.9% ± 2.0% (n=227)	4.6% ± 0.0% (n=5371)
97%	5.2% ± 2.0% (n=155)	6.2% ± 1.0% (n=2186)	6.1% ± 2.0% (n=294)	4.0% ± 0.0% (n=6514)
98%	3.7% ± 2.0% (n=206)	5.7% ± 1.0% (n=2843)	3.8% ± 1.0% (n=362)	3.3% ± 0.0% (n=7833)
99%	3.9% ± 1.0% (n=340)	4.5% ± 0.0% (n=3510)	3.8% ± 1.0% (n=586)	2.7% ± 0.0% (n=11220)
100%	1.0% ± 0.0% (n=633)	3.4% ± 0.0% (n=9854)	4.7% ± 1.0% (n=286)	1.1% ± 0.0% (n=9504)

© 2021 Wong AI et al. *JAMA Network Open.*

eTable 10. Characterization of the Distribution of Hidden Hypoxemia by Respiratory SOFA Score

To determine the occurrence of hidden hypoxemia by rSOFA score, hidden hypoxemia is stratified for all PhysioNet patients (n=41,590).

The Chi-square test of homogeneity demonstrates p < 5e-37. This results shows that the occurrence of hidden hypoxemia is not similar across rSOFA patient subgroups, suggesting that rSOFA and hidden hypoxemia are complementary indicators of a patient's respiratory function status. Although the likelihood of hidden hypoxemia could be thought to increase with rSOFA, this relationship does not seem to be linear. However, the occurrence of hidden hypoxemia is the highest (12.7%) among patients who have the highest rSOFA (equal to 4) at baseline (i.e., at the time of ABG).

rSOFA	Hidden hypoxemia (n)	No hypoxemia (n)	Total per rSOFA category (n)	Observed hidden hypoxemia (%)	Expected hidden hypoxemia (%)
**0**	1718	23357	25075	6.9%	6.3%
**1**	58	1999	2057	2.8%	6.3%
**2**	531	9008	9539	5.6%	6.3%
**3**	142	3500	3642	3.9%	6.3%
**4**	162	1115	1277	12.7%	6.3%
**Total**	2611	38979	41590		

© 2021 Wong AI et al. *JAMA Network Open.*

eTable 11. Distribution of In-Hospital Mortality, Stratified by Respiratory SOFA Score and Presence of Hidden Hypoxemia

This table describes the risk of in-hospital mortality, stratified by both the rSOFA score and the presence of hidden hypoxemia. Regardless of the rSOFA, presence of hidden hypoxemia is associated with a higher risk of in-hospital mortality. We have stratified the distribution of in-hospital mortality by rSOFA alone and by both hidden hypoxemia and rSOFA together with hidden hypoxemia. The relationship between mortality and hidden hypoxemia is not similar across rSOFA patient subgroups, irrespective of the status of patients with missing information (Chi-square test for homogeneity: $p < 1.2e-5$ assuming patients with missing data lived, $p < 1.5e-5$ assuming patients with missing data died). In terms of mortality outcomes, rSOFA and hidden hypoxemia seem to provide complementary information so that the combination of rSOFA and hidden hypoxemia is a better predictor of mortality than rSOFA alone. Our results demonstrate that the presence of hidden hypoxemia at any rSOFA score is associated with higher mortality: while at a rSOFA of 4, experiencing hidden hypoxemia increased the likelihood of death by ~4% in the five studied datasets, this probability reached up to ~16% at rSOFA = 2.

	Hidden hypoxemia				No hypoxemia				All			
rSOFA	*n*	died	lived	missing	*n*	died	lived	missing	*n*	died	lived	missing
0	*1718 (65.8%)*	417 (24.3%)	1288 (75%)	13 (0.8%)	*1288 (68.8%)*	4050 (17.3%)	19079 (81.7%)	228 (1%)	*25075 (60.3%)*	4467 (17.8%)	20367 (81.2%)	241 (1%)
1	*58 (2.2%)*	16 (27.6%)	40 (69%)	2 (3.4%)	*40 (2.1%)*	283 (14.2%)	1698 (84.9%)	18 (0.9%)	*2057 (4.9%)*	299 (14.5%)	1738 (84.5%)	20 (1%)
2	*531 (20.3%)*	179 (33.7%)	350 (65.9%)	2 (0.4%)	*350 (18.7%)*	1458 (16.2%)	7486 (83.1%)	64 (0.7%)	*9539 (22.9%)*	1637 (17.2%)	7836 (82.1%)	66 (0.7%)
3	*142 (5.4%)*	50 (35.2%)	91 (64.1%)	1 (0.7%)	*91 (4.9%)*	747 (21.3%)	2739 (78.3%)	14 (0.4%)	*3642 (8.8%)*	797 (21.9%)	2830 (77.7%)	15 (0.4%)
4	*162 (6.2%)*	59 (36.4%)	102 (63%)	1 (0.6%)	*102 (5.5%)*	352 (31.6%)	759 (68.1%)	4 (0.4%)	*1277 (3.1%)*	411 (32.2%)	861 (67.4%)	5 (0.4%)
Total	*2611*	721 (27.6%)	1871 (71.7%)	19 (0.7%)	*1871*	6890 (17.7%)	31761 (81.5%)	328 (0.8%)	*41590*	7611 (18.3%)	33632 (80.9%)	347 (0.8%)

© 2021 Wong AI et al. *JAMA Network Open.*

Scenario 1: all patients with missing information lived

a) Testing the relationship between rSOFA, hidden hypoxemia, and in-hospital mortality ($p < 1.2e-5$)

rSOFA	Died and had hidden hypoxemia (n)	Died and had no hypoxemia (n)	Total per rSOFA category (n)	Observed mortality and hidden hypoxemia (%)	Expected mortality and hidden hypoxemia (%)	Expected mortality and hidden hypoxemia (n)
**0**	417	4050	4467	9.3%	9.5%	423.2
**1**	16	283	299	5.4%	9.5%	28.3
**2**	179	1458	1637	10.9%	9.5%	155.1
**3**	50	747	797	6.3%	9.5%	75.5
**4**	59	352	411	14.4%	9.5%	38.9
**Total**	721	6890	7611			

<The remainder of this page intentionally left blank.>

© 2021 Wong AI et al. *JAMA Network Open.*

b) Assessing whether the combination of rSOFA and hidden hypoxemia is a better signal / predictor of mortality than rSOFA alone

i) In-hospital mortality stratified by rSOFA alone ($p < 3.9e-39$)

rSOFA alone	Died (n)	Alive (n)	Total (n)	Observed mortality (%)	Expected mortality (%)	Expected mortality (n)
**0**	4467	20608	25075	17.8%	18.3%	4588.7
**1**	299	1758	2057	14.5%	18.3%	376.4
**2**	1637	7902	9539	17.2%	18.3%	1745.6
**3**	797	2845	3642	21.9%	18.3%	666.5
**4**	411	866	1277	32.2%	18.3%	233.7
**Total**	7611	33979	41590			

ii) In-hospital mortality stratified by rSOFA + hidden hypoxemia ($p < 2.4e-4$)

rSOFA + hidden hypoxemia	Died (n)	Alive (n)	Total (n)	Observed mortality (%)	Expected mortality (%)	Expected mortality (n)
**0**	417	1301	1718	24.3%	27.6%	474.4
**1**	16	42	58	27.6%	27.6%	16.0
**2**	179	352	531	33.7%	27.6%	146.6
**3**	50	92	142	35.2%	27.6%	39.2
**4**	59	103	162	36.4%	27.6%	44.7
**Total**	721	1890	2611			

© 2021 Wong AI et al. *JAMA Network Open.*

<u>Scenario 2:</u> all patients with missing information died

c) Testing the relationship between rSOFA, hidden hypoxemia, and in-hospital mortality ($p < 1.5e\text{-}5$)

rSOFA	Died and had hidden hypoxemia (n)	Died and had no hypoxemia (n)	Total per rSOFA category (n)	Observed mortality and hidden hypoxemia (%)	Expected mortality and hidden hypoxemia (%)	Expected mortality and hidden hypoxemia (n)
**0**	430	4278	4708	9.1%	9.3%	437.8
**1**	18	301	319	5.6%	9.3%	29.7
**2**	181	1522	1703	10.6%	9.3%	158.4
**3**	51	761	812	6.3%	9.3%	75.5
**4**	60	356	416	14.4%	9.3%	38.7
**Total**	740	7218	7958			

<The remainder of this page intentionally left blank.>

© 2021 Wong AI et al. *JAMA Network Open.*

d) Assessing whether the combination of rSOFA and hidden hypoxemia is a better signal / predictor of mortality than rSOFA alone

i) In-hospital mortality stratified by rSOFA alone ($p < 2.5e-34$)

rSOFA alone	Died (n)	Alive (n)	Total (n)	Observed mortality (%)	Expected mortality (%)	Expected mortality (n)
**0**	4708	20367	25075	18.8%	19.1%	4798.0
**1**	319	1738	2057	15.5%	19.1%	393.6
**2**	1703	7836	9539	17.9%	19.1%	1825.2
**3**	812	2830	3642	22.3%	19.1%	696.9
**4**	416	861	1277	32.6%	19.1%	244.3
**Total**	7958	33632	41590			

ii) In-hospital mortality stratified by rSOFA + hidden hypoxemia ($p < 4.7e-4$)

rSOFA + hidden hypoxemia	Died (n)	Alive (n)	Total (n)	Observed mortality (%)	Expected mortality (%)	Expected mortality (n)
**0**	430	1288	1718	25.0%	28.3%	486.9
**1**	18	40	58	31.0%	28.3%	16.4
**2**	181	350	531	34.1%	28.3%	150.5
**3**	51	91	142	35.9%	28.3%	40.2
**4**	60	102	162	37.0%	28.3%	45.9
**Total**	740	1871	2611			

© 2021 Wong AI et al. *JAMA Network Open.*

eTable 12. Descriptive Statistics for Patients With Hidden Hypoxemia vs Patients Without Hypoxemia, Carboxyhemoglobin Less than 2 and Methemoglobin Less Than 2 (n = 36,843)

For patients with recorded carboxyhemoglobin < 2 and methemoglobin < 2, the table presents baseline patient demographic (age, sex) and clinical characteristics (SOFA, CVSOFA) at the time of ABG, stratified by race/ethnicity. When applicable, standard errors (SE) are provided. These were obtained using simple bootstrap with 100 iterations.

	Asian			Black			Hispanic			White		
	Hidden hypoxemia	No hypoxemia	p	Hidden hypoxemia	No hypoxemia	p	Hidden hypoxemia	No hypoxemia	p	Hidden hypoxemia	No hypoxemia	p
n	33 (3.3%)	958 (96.7%)		817 (4.9%)	15874 (95.1%)		72 (4.1%)	1670 (95.9%)		414 (2.4%)	17005 (97.6%)	
In-hospital mortality	6 (18.2%)	141 (14.7%)	0.58	156 (19.1%)	2293 (14.4%)	< 0.001	14 (19.4%)	287 (17.2%)	0.58	89 (21.5%)	2045 (12%)	< 0.001
Female	13 (39.4%)	368 (38.4%)		401 (49.1%)	7708 (48.6%)		39 (54.2%)	682 (40.8%)		194 (46.9%)	6616 (38.9%)	
Male	20 (50%)	590 (50%)		416 (50%)	8166 (50%)		33 (50%)	988 (50%)		220 (50%)	10389 (50%)	
Age	69.06 ± 3.18	62.41 ± 0.64	< 0.001	59.57 ± 0.68	58.53 ± 0.17	< 0.001	63.98 ± 2.6	60.35 ± 0.55	< 0.001	64.29 ± 1.02	63.24 ± 0.15	< 0.001
Length of Stay (days)	16.94 ± 3.69	14.42 ± 0.7	< 0.001	13.2 ± 0.71	16.34 ± 0.19	< 0.001	12.67 ± 1.96	13.98 ± 0.49	< 0.001	12.63 ± 0.87	14.22 ± 0.18	< 0.001
SaO2	82.37 ± 1.37	97.05 ± 0.12	< 0.001	79.54 ± 0.51	96.8 ± 0.03	< 0.001	80.5 ± 1.31	96.66 ± 0.09	< 0.001	82.43 ± 0.52	96.99 ± 0.03	< 0.001
SpO2	93.95 ± 0.87	98.0 ± 0.11	< 0.001	94.99 ± 0.19	97.92 ± 0.03	< 0.001	94.89 ± 0.59	97.42 ± 0.09	< 0.001	93.39 ± 0.25	97.56 ± 0.03	< 0.001

© 2021 Wong AI et al. *JAMA Network Open.*

CVSOFA		2.44 ± 0.79	< 0.001	1.73 ± 0.65	1.24 ± 0.15	< 0.001	1.63 ± 0.35	1.21 ± 0.07	< 0.001	1.43 ± 0.32	1.24 ± 0.07	< 0.001
RSOFA		1.16 ± 0.75	< 0.001	1.43 ± 0.63	0.96 ± 0.13	< 0.001	0.94 ± 0.26	1.06 ± 0.06	< 0.001	1.28 ± 0.31	0.98 ± 0.06	< 0.001
SOFA		8.88 ± 1.9	< 0.001	7.6 ± 1.34	5.83 ± 0.45	< 0.001	5.81 ± 0.75	5.61 ± 0.19	0.0121	6.78 ± 1.01	5.37 ± 0.21	< 0.001
Future SOFA		8.18 ± 0.68	< 0.001	9.3 ± 1.46	7.3 ± 0.47	< 0.001	8.62 ± 0.88	7.0 ± 0.18	< 0.001	9.3 ± 1.13	6.74 ± 0.23	< 0.001
Future CVSOFA		2.39 ± 0.78	< 0.001	2.44 ± 0.63	1.54 ± 0.14	< 0.001	2.32 ± 0.31	1.58 ± 0.07	< 0.001	2.27 ± 0.37	1.55 ± 0.08	< 0.001
Future RSOFA		1.17 ± 0.59	< 0.001	2.75 ± 0.62	1.68 ± 0.16	< 0.001	2.45 ± 0.27	1.73 ± 0.07	< 0.001	2.39 ± 0.34	1.63 ± 0.08	< 0.001
Serum creatinine												
Before ABG	1.74 ± 0.43	1.61 ± 0.08	0.0335	2.53 ± 0.15	2.46 ± 0.03	< 0.001	1.79 ± 0.32	1.71 ± 0.08	0.1192	1.58 ± 0.1	1.41 ± 0.01	< 0.001
24 hours after ABG	1.89 ± 0.46	1.4 ± 0.07	< 0.001	2.31 ± 0.12	2.1 ± 0.02	< 0.001	2.12 ± 0.51	1.51 ± 0.07	< 0.001	1.46 ± 0.12	1.29 ± 0.01	< 0.001
Difference	-0.01 ± 0.27	-0.18 ± 0.05	< 0.001	-0.21 ± 0.1	-0.34 ± 0.02	< 0.001	-0.24 ± 0.34	-0.22 ± 0.05	0.3538	-0.07 ± 0.08	-0.09 ± 0.01	< 0.001
Serum lactate												
Before ABG	2.75 ± 0.61	2.95 ± 0.12	< 0.001	2.93 ± 0.17	2.72 ± 0.03	< 0.001	2.63 ± 0.61	2.78 ± 0.09	< 0.001	2.82 ± 0.23	2.61 ± 0.03	< 0.001
24 hours after ABG	1.96 ± 0.39	2.35 ± 0.19	< 0.001	2.84 ± 0.3	2.38 ± 0.04	< 0.001	3.23 ± 1.31	2.16 ± 0.18	< 0.001	2.34 ± 0.37	2.11 ± 0.04	< 0.001
Difference	-0.94 ± 1.04	-1.08 ± 0.23	0.0045	-0.24 ± 0.3	-0.68 ± 0.06	< 0.001	1.28 ± 1.77	-0.94 ± 0.23	< 0.001	-0.53 ± 0.39	-0.84 ± 0.07	< 0.001

© 2021 Wong AI et al. *JAMA Network Open.*
